# KIFC3 Promotes Proliferation, Migration, and Invasion in Colorectal Cancer *via* PI3K/AKT/mTOR Signaling Pathway

**DOI:** 10.3389/fgene.2022.848926

**Published:** 2022-06-22

**Authors:** Huiling Liao, Lan Zhang, Shimin Lu, Wei Li, Weiguo Dong

**Affiliations:** ^1^ Department of Gastroenterology, Renmin Hospital of Wuhan University, Wuhan, China; ^2^ Central Laboratory, Renmin Hospital of Wuhan University, Wuhan, China

**Keywords:** KIFC3, proliferation, migration, invasion, epithelial-to-mesenchymal transition, PI3K/Akt/mTOR signal pathway

## Abstract

**Background:** KIFC3, belongs to kinesin superfamily proteins (KIFs), is well known for its role in intracellular cargo movement. KIFC3 has been identified as a docetaxel resistance gene in breast cancer cells, however, the role of KIFC3 and its potential mechanism in colorectal cancer (CRC) remains elusive.

**Objectives:** We aims to investigate the effects of KIFC3 in proliferation, migration, and invasion in CRC as well as the potential mechanism inside.

**Methods:** We investigated the expression of KIFC3 in the Oncomine, Gene Expression Profiling Interactive Analysis databases. The KIFC3 protein expression and mRNA level in CRC cells were evaluated by western blot and qRT-PCR. Cell proliferation ability was detected by CCK-8, EdU, colony formation assay and xenograft tumor in nude mice. Flow cytometry was used to detect the cell cycle. The effect of KIFC3 on the epithelial-to-mesenchymal transition (EMT) was investigated by transwell and wound healing assay. The association of KIFC3 with EMT and PI3K/AKT/mTOR signaling pathway were measured by western blot and immunofluorescence staining.

**Results:** The expression of KIFC3 was higher in CRC tissues than normal colorectal tissue, and was negatively correlated with the overall survival of patients with CRC. KIFC3 silencing inhibited the proliferation, migration and invasion of CRC cells. Meanwhile, it could decrease the number of cells in S phase. KIFC3 silencing inhibited the expression of proliferating cell nuclear antigen, Cyclin A2, Cyclin E1, and CDK2 and increased the expression of p21 and p53. KIFC3 overexpression promoted the G1/S phase transition. KIFC3 silencing inhibited the EMT process, which decreased the level of N-cadherin, Vimentin, SNAIL 1, TWIST, MMP-2, MMP-9 and increased E-cadherin, while KIFC3 overexpression show the opposite results. Furthermore, the knockdown of KIFC3 suppressed the EMT process by modulating the PI3K/AKT/mTOR signaling pathway. KIFC3 silencing decreased the expression of phosphorylated PI3K, AKT, mTOR, but total PI3K, AKT, mTOR have no change. Inversely, the upregulation of KIFC3 increased the expression of phosphorylated PI3K, AKT and mTOR, total PI3K, AKT, mTOR have no change. In a xenograft mouse model, the depletion of KIFC3 suppressed tumor growth. the increased expression levels of KIFC3 could enhance the proliferation, migration and invasion of CRC cells, and enhance the EMT process through the PI3K/AKT/mTOR pathway.

**Conclusion:** Our study substantiates that KIFC3 can participate in the regulation of CRC progression by which regulates EMT *via* the PI3K/AKT/mTOR axis.

## Introduction

Colorectal cancer (CRC) is one of the most prevalent malignancies in the digestive system. The incidence of CRC is rising year after year, and CRC ranks third in terms of incidence, but second in terms of mortality (Siegel et al., 2020; Sung et al., 2021). CRC is an important barrier to increasing life expectancy in every country of the world, which seriously threatens human health (Dekker et al., 2019; Sung et al., 2021). Despite that there have been great improvements in CRC treatment in the clinic, and that comprehensive therapy has partly prolonged the survival rate, the prognosis of CRC patients was still not satisfied, as well as that the 5-year survival rate of patients with CRC remains low (Chen et al., 2014; Ishikawa et al., 2019). Most patients are diagnosed at an advanced stage, and accompanied by metastasis, which limits their options for therapeutic strategies (Zhou et al., 2019; Hao et al., 2021). Moreover, the postoperative recurrence rate and metastasis remain high (Li et al., 2020a). Hence, there is an urgent need to seek novel, sensitive and specific biomarkers to improve CRC prognosis and therapeutic targets for CRC.

Intracellular organelle transport is essential for cell morphogenesis, promoting cell survival and maintaining the function of the cell. Among the molecular motors that are involved in intracellular transport, three large superfamilies have been identified—kinesins, dyneins and myosins (Hirokawa and Tanaka, 2015). The Kinesin Superfamily Proteins (KIFs) is a group of proteins that share a highly conserved motor domain, which have been shown to transport organelles, protein complexes, and mRNAs to specific destinations in a microtubule- and ATP dependent manner and also participate in chromosomal and spindle movements during mitosis and meiosis (Miki et al., 2003; Liu et al., 2013). KIFs consist of 45 family members, which play different roles in the genesis and development of tumors (Seog et al., 2004; Hirokawa and Tanaka, 2015). A number of KIFs show aberrant overexpression in various cancer cells, such as KIF4A, which is upregulated in cervical cancer and lung cancer, inhibits the repair of damaged DNA double-strand in lung cancer cells sensitive to cisplatin (Wan et al., 2019). KIF20B is overexpressed in bladder cancer and is considered as a cancer-testis antigen specific to human bladder cancer (Kanehira et al., 2007; Liu et al., 2013). KIF2A is overexpressed in squamous cell carcinoma of the oral tongue, and the downregulation of KIF2A can induce apoptosis in squamous cell carcinoma of the oral tongue through the inhibition of the PI3K/AKT signaling pathway (Wang et al., 2014). KIF18B is highly expressed in colon adenocarcinoma tissues and negatively correlated with patients’ prognosis (Zhao et al., 2020).

The kinesin family member C3 (KIFC3) gene, is located on the human chromosome 16q13-q21 (Hoang et al., 1998). KIFC3 has minus end–directed microtubule motor activity and functions in golgi localization, integration and apical transport of epithelial cells (Noda et al., 2001; Xu et al., 2002). KIFC3 has been identified as a docetaxel resistance gene in breast cancer cells (Tan et al., 2012; Li and Bakhoum, 2019). Besides, KIFC3 was also found to be associated with hepatocellular carcinoma. The high expression of KIFC3 suggests shorter survival time, and KIFC3 is associated with migration and invasion of hepatocellular carcinoma (Li et al., 2017). However, our knowledge of its function in CRC is still limited.

In this study, we determined whether KIFC3 will have an effect in the progression of CRC on the proliferation, migration and invasion. Then, we performed subcutaneous tumor formation experiments to verify the effects of KIFC3. Furthermore, we explored the potential mechanism by which KIFC3 is involved in the process with a focus on the regulation of the PI3K/AKT/mTOR signaling pathway.

## Materials and Methods

### Gene Expression Data From Public Databases

We analyzed KIFC3 mRNA levels in CRC and normal colorectal tissues using the Oncomine (http://www.oncomine.org) and GEPIA (http://gepia.cancer-pku.cn/) databases. The GEPIA database was used to analyze the information in The Cancer Genome Atlas (TCGA) database (https://tcga-data.nci.nih.gov/tcga/) to evaluate the prognostic value of KIFC3 level. Raw counts of RNA-sequencing data and corresponding clinical information from KIFC3 in CRC were obtained from International Cancer Genome Consortium (ICGC) database (https://dcc.icgc.org/releases/current/Projects). The Kaplan–Meier survival analysis with log-rank test were also used to compare the survival difference between above two groups. Time-ROC analysis was performed to compare the predictive accuracy of each gene and risk score. For Kaplan–Meier curves, *p*-values and hazard ratio (HR) with 95% confidence interval (CI) were generated by log-rank tests and univariate Cox proportional hazards regression. All analytical methods above and R packages were performed using R software version v4.0.3 (The R Foundation for Statistical Computing, 2020). *p* < 0.05 was considered as statistically significant. The Kyoto Encyclopedia of Genes and Genomes (KEGG) and Gene Ontology (GO) enrichment analysis was performed with KIFC3-associated genes using the clusterProfiler package in R (https://bioconductor.org/packages/clusterProfiler). FDR *p* < 0.05 is used to distinguish significant enrichment items. Enter the gene and use the “enrichment GO” and “enrichment KEGG” functions for enrichment analysis. The bubble chart, bar chart and network chart are used to show the results of the enrichment analysis.

### Cell Culture

Human colorectal cancer cell lines (HT29, HCT116, SW480, DLD-1 cell) and the normal intestinal epithelial cell line (NCM460) were purchased from the China Center for Type Culture Collection (Wuhan, China). These cells were cultured in Roswell Park Memorial Institute (RPMI) 1640 medium (HyClone, United States) containing 10% heat-inactivated fetal bovine serum (FBS, Gibco, United States) and 1% penicillin-streptomycin (Gibco, United States) under a humidified atmosphere incubator of 5% CO_2_ at 37°C.

### Antibodies and Reagents

Antibodies against KIFC3 were purchased from Abcam (Cambridge, MA, United States). Antibodies against proliferating cell nuclear antigen (PCNA), matrix metalloproteinase (MMP)-2, MMP-9, glyceraldehyde-3-phosphate dehydrogenase (GAPDH), p21, Cyclin A2, Cyclin E1, CDK2, E-cadherin, N-cadherin, Vimentin, Snai1 were purchased from Proteintech (Rosemont, IL, United States). Antibodies against p53, TWIST1 were obtained from Affinity (Cincinnati, United States). Antibodies against total phosphatidylinositol 3-kinase (t-PI3K), phosphorylated phosphatidylinositol 3-kinase (p-PI3K), total AKT (t-AKT), phosphorylated Akt (p-AKT), total mammalian target of rapamycin (t-mTOR), phosphorylated mammalian target of rapamycin (p-mTOR) were obtained from Cell Signaling Technology (Danvers, MA, United States). Antibodies against PCNA, GAPDH, E-cadherin, N-cadherin, Vimentin, were used at a working concentration of 1:5000, and the other antibodies were used at a working concentration of 1:1000 and were stored at 4°C. The secondary antibodies were purchased from Proteintech (Rosemont, IL, United States) and used at a dilution ratio of 1:10,000.

### Lentivirus Vectors and Transfection

Three pairs of short hairpin RNA (shRNA) targeting KIFC3 lentivirus vectors (shKIFC3#1, shKIFC3#2, shKIFC3#3) and negative controls (NC) that contain green fluorescent protein (GFP) and purinomycin resistance were purchased from Shanghai Genechem Company (Shanghai, China). The cells were assigned as follows: control group (SW480 or HT29 cells), negative control (NC) group (SW480 or HT29 cell transfected with blank plasmid), shKIFC3 group (SW480 or HT29 cell transfected shKIFC3). SW480 and HT29 cells silenced stably for KIFC3 expression were generated using lentiviral constructs expressing shKIFC3 and negative control. The encoding sequence of KIFC3 was cloned into lentivirus GV248 vector, and the component sequence was hU6-MCS-Ubiquitin-EGFP-IRES-puromycin. Transfection was performed in accordance with the manufacturer’s instructions. SW480 and HT29 cells in logarithmic growth phase were collected and inoculated in 6-well plates after digestion with pancreatin. The density of inoculation was 5 × 10^4^ cells/ml, and the cells were cultured overnight in a CO_2_ incubator with a volume fraction of 5% at 37°C. According to the instructions, and as growth reached approximately 60%, a RPMI 1640 medium containing lentivirus was added at a multiplicity of infection (MOI) of 100 and mixed with the cells. We use HiTransG A to improve infection efficiency. Also, the overexpression of KIFC3 lentivirus and control vector, which contains red fluorescent protein (RFP) and purinomycin resistance, infected the DLD-1 cell respectively. The KIFC3 lentivirus and control vector were synthesized by Shanghai Genechem Company (Shanghai, China). Similarly, as the procedure described above, DLD-1 cells were transfected with lentivirus vectors carrying OVER or Vector, respectively. We added puromycin (Thermo Fisher Scientific, Waltham, United States) to the medium to screen stable transfected cell lines. The efficiency of knockdown or overexpression were measured at 48 h post-transfection by western blotting and qPCR. The vector with the highest knockdown efficiency was selected for subsequent experiments.

### Total RNA Extraction and Quantitative Real-Time PCR

TRIzol reagent (Takara, Japan) was used to extract total RNA from cell lysates, according to the manufacturer’s instructions. We used the NanoDrop™ One (Thermo Fisher Scientific, United States) to determine the RNA concentration and purity. Total RNA was reverse transcribed into cDNA using the PrimeScriptTMRT reagent kit with gDNAEraser (Perfect Real Time, Takara, Japan). Target gene mRNA expression levels were detected using TB Green® Premix Ex Taq™ II (TliRNaseH Plus, Takara, Japan). KIFC3 mRNA level was measured by qPCR with CFX Connect Real-Time System (Bio-Rad, United States) and the β-actin was used as an internal reference. The primers sets are as follows: KIFC3 forward, 5′-GCA​GAT​TGC​CAT​GTA​CGA​GTC-3′; reverse, 5′- CGG​ACG​CCT​GCT​AGA​TTC​TC -3′, β-actin forward, 5′-GCA​CAG​GGT​GCT​CCT​CAG-3′; reverse primer 5′-CTA​GGC​ACC​AGG​GTG​TGA​TG-3′. The data were calculated by the 2^−ΔΔCt^ method. All qPCR reactions were run in triplicates.

### Western Blot Analysis

The proteins in CRC cells lines (HT29, HCT116, SW480, DLD-1 cell) and the NCM460 cell were extracted from the cell lysates using Radio Immunoprecipitation Assay (RIPA) buffer (Biyotime, China) which containing phosphatase inhibitors and protease inhibitor cocktail, and the concentration of protein was tested with a bicinchoninic acid (BCA) protein assay kit (Biyotime, China) following the instructional manual. Equal mass of protein was separated by SDS-polyacrylamide gel electrophoresis (10% or 8%) and then transferred onto 0.45 µm polyvinylidene difluoride (PVDF, Millipore, United States) membranes. All the PVDF membranes were incubated in Tris-Buffered Saline with 1% TWEEN 20 (TBST, Cell Signaling Technology) blocking solution containing 5% skim milk or 5% Bovine Serum Albumin (BSA) at room temperature for 1–2 h. Next, the membranes were washed on a shaker for 3 × 10 min using TBST. The primary antibodies were added and incubated at 4°C, overnight. Then, after being washed with TBST three times, the membranes were incubated with horseradish peroxidase (HRP)-conjugated secondary antibodies Proteintech (Rosemont, IL, United States) for 1 h at room temperature and washed with TBST again. Finally, the protein bands were detected by ChemiDocTM Touch (Bio-Rad, United States).

### Cell Counting Kit-8 Assay

Cell proliferation was examined using the Cell Counting Kit-8 (CCK-8, Beyotime, Shanghai, China) assay. SW480, HT29, and DLD-1 (at 4× 10^3^ cells/well) were resuspended and seeded in 96-well culture plates, and allowed to attach overnight in complete growth medium at 37°C with 5% CO_2_. The supernatant was removed, 10 µl of CCK-8 was added to each well and incubated for 2 h. After 2 h of incubation, the absorbance of the colored formazan reaction product was evaluated at 450 nm by a microplate reader (Perkin Elmer, United States). We measured when the cells were cultured for 24, 36 and 48 h, respectively. All experiments were performed in triplicate to determine their reproducibility.

### 5-Ethynyl-2-deoxyuridine Assay

According to the operation instructions of the EdU assay kit (Beyotime, Shanghai, China), the transfected cells (at 1 × 10^4^ cells/well) were seeded into 24-well plates for 24 h. Then, add 1 ml culture solution containing EdU to each well, and incubate at 37°C for 2 h. Adding 1 ml of 4% paraformaldehyde solution (Servicebio, China) to fix the fine cells for 15 min; after being washed with Phosphate-buffered saline (PBS), cells were permeabilized with 0.3% TritonX-100 (Biotech, China) for 10 min at room temperature. Cells were washed with PBS three times. Subsequently, cells were reacted with Apollo reaction mixture for 30 min. Then cells were washed 3 times using PBS. Cell nuclei were stained with Hoechst 33,258 to keep away from light for 10 min. After being washed by PBS, the proliferation of cells was visualized under a fluorescence microscope (Olympus BX51, Japan). The percentage of the EdU-positive cells and the average fluorescence intensity were calculated by Image J software (NIH, United States). Each group repeated the process thrice.

### Colony Formation Assay

Colony forming ability was detected with colony formation assay. The cell suspensions of SW480, HT29, and DLD-1 (at 1 × 10^3^ cells/well) were plated into 6-well plates and incubated at 37°C and at an atmosphere of 5% CO_2_ for 7–10 days. The supernatants were discarded, and the colonies washed with PBS for three times, and fixed in 4% paraformaldehyde solution (Servicebio, China) to fix the cells for 15 min. Next, cells were stained with 0.2% crystal violet at room temperature for 10 min. Finally, the plate was washed moderately with running tap water. The number of colonies containing more than 50 cells was microscopically counted to calculate the colony formation rate as the number of colonies/number of cells × 100% divided by control. The data was analyzed by Prism software 7.0 (GraphPad Software, Inc.). All experiments were performed in triplicate to determine their reproducibility.

### Wound Healing Assay

The cell suspensions of SW480, HT29, and DLD-1 (at 1×10^5^ cells/well) were placed into a 6-well plates chamber and incubated in RPMI 1640 medium at 37°C with 5% CO_2_. When the cells reached 90% confluence, a 200-µl pipette tip was used to scratch shaped wounds consistently on the cell monolayer across each well. Then, PBS was utilized to remove floating cells, and serum-free RPMI 1640 medium was added. The wound was photographed immediately (0 h), using an inverted optical microscope (×200) (Olympus IX51, Japan). The cells were then cultured in the medium. The wounds were photographed at 48 h to measure the extent of wound healing. The change in the scratch area with time and the wound healing percent were calculated as follows: Wound healing (%)  = (the initial scratch area—the scratch area after 48 h)/the initial scratch area × 100%. The process above was conducted by Image J. Each experiment was repeated 3 times.

### Flow Cytometry

Cell cycle analysis was found using flow cytometry. The cells growing in exponential phase were seeded into 6-well plates cells and treated for 48 h. Next, cells were digested by 0.25% trypsin without EDTA, and then harvested and resuspended in one volume of PBS. Following treatment, cells were fixed with ice-cold 75% ethanol at 4°C overnight. The fixed cell pellets then centrifuged at 1,200 rpm for 10 min. With the removal of the supernatants, cells precipitation was suspended in 1 ml propidium iodide (PI, Antgene, China) staining solution (50 μg/ml PI and 100 μg/ml RNase A in PBS) in darkness before flow cytometric analysis. After incubating for 30 min, the percentages of cells in the G0/G1, S, and G2/M cell cycle phases were analyzed by flow cytometry (FACS Calibur, Becton Dickinson, Franklin Lakes, NJ, United States). Data from at least three independent experiments were analyzed using the Modfit LT^TM^ Software.

### Immunofluorescence

Cells were cultured on coverslips (14 mm) in 24-well plates at 37°C with 5% CO_2_. After 24 h, the supernatant was removed and PBS was used to wash it. Then, add 1 ml 4% formaldehyde to each well for 15 min. After being washed by PBS, cells were permeabilized with 0.3% Triton X-100 at room temperature for 10 min. Then, blocked with 5% BSA for 30 min and washed by PBS three times. After that, the washed cells were incubated with polyclonal antibodies against E-cadherin (Proteintech, 1: 50), N-cadherin (Proteintech, 1: 50), p-PI3K (Cell Signaling Technology, 1: 400), and p-AKT (Cell Signaling Technology 1: 400), overnight at 4°C in darkness. After being washed with PBS, add FITC-labeled and Cy3-labeled secondary antibody, 1:500 dilution, for 1 h at dark. DAPI (Biosharp Biotech, Hefei, China, 1: 1000) was used to counterstain the nuclei for 10 min. The slides were mounted on coverslips with an anti-fade mounting medium. All the images were collected by an upright fluorescence microscope (Olympus BX51, Japan). Each experiment was repeated 3 times.

### Transwell Migration and Invasion Assay

Cell migration and invasion ability were examined by Corning transwell insert chambers (8 mm pore size, Corning). The transwell chamber was placed into the 24-well culture plate, the chamber was called the upper chamber, and the culture plate was called the lower chamber. In the migration assay, 2 × 10^5^ cells were suspended in 100 μl FBS-free medium and were added to the upper chamber. 600 μl RPMI 1640 with 20% FBS were seeded in the lower compartment of the chamber and the cells were incubated in an environment of 37°C and 5% CO_2_. After 24 h, 4% paraformaldehyde (Servicebio, China) was used for fixation. Afterwards, 0.5% crystal violet was dyed for 10 min and the membrane was washed with PBS. The migrated cells on the lower surface were photographed randomly with an Olympus IX51 inverted microscope in five visual fields (× 200 magnification) and the migrated cells were quantified using Image J software. For invasion assay, the upper compartment was precoated with one hundred microliters of matrigel and 5 × 10^5^ cells per invasion well were added to the upper chamber. After incubating for 48 h, the rest of the protocol was similar to the migration assay. Each assay was performed at least three times.

### Xenograft Tumor Model

5-week-old male BALB/c nude mice were purchased from Beijing HFK Experimental Animal Center (Beijing, China). SW480 cells stably transfected with shKIFC3 and shNC were suspended in PBS, about 200 μl of cells suspension were subcutaneously injected into the lower right dorsal flank of all mice. Each group included seven mice. Tumor formation was observed and tumor volume was measured by the vernier caliper on day 7 after injection of cell suspension. Then the growth of tumors was monitored for an interval of 1 day. The tumor volume was calculated at the same time intervals, according to the formula: volume (mm^3^) = 0.5 × longest diameter × (shortest diameter)^2^. When the tumors reached a minimal diameter of 1.0 cm, the subcutaneous tumors were dissected and then measured and weighted after the sacrifice of the mice. The tumor sections were removed from paraffin-embedded blocks of the harvested tissues and we used KIFC3 and ki67 antibodies on the sections for IHC. All animal experiments were performed following the procedures and principles outlined in the National Institutes of Health guidelines for the care and use of laboratory animals, and were approved by the Animal Care and Use Committee of Renmin Hospital of Wuhan University.

### Statistical Analysis

All data was expressed as mean ± standard deviation (SD). All statistical analyses were performed using GraphPad Prism 8.0. Comparisons between two groups were determined using Student t-test, while comparisons between multiple groups were analyzed using one-way ANOVA followed by Tukey’s post-hoc tests. Overall survival (OS) and disease-free survival (DFS) curves was calculated by Kaplan-Meier method and tested by log-rank test. *p* values <0.05 were considered statistically significant.

## Results

### Kinesin Family Member C3 mRNA Levels Are Significantly Upregulated in Colorectal Cancer Tissues

**FIGURE 1:**
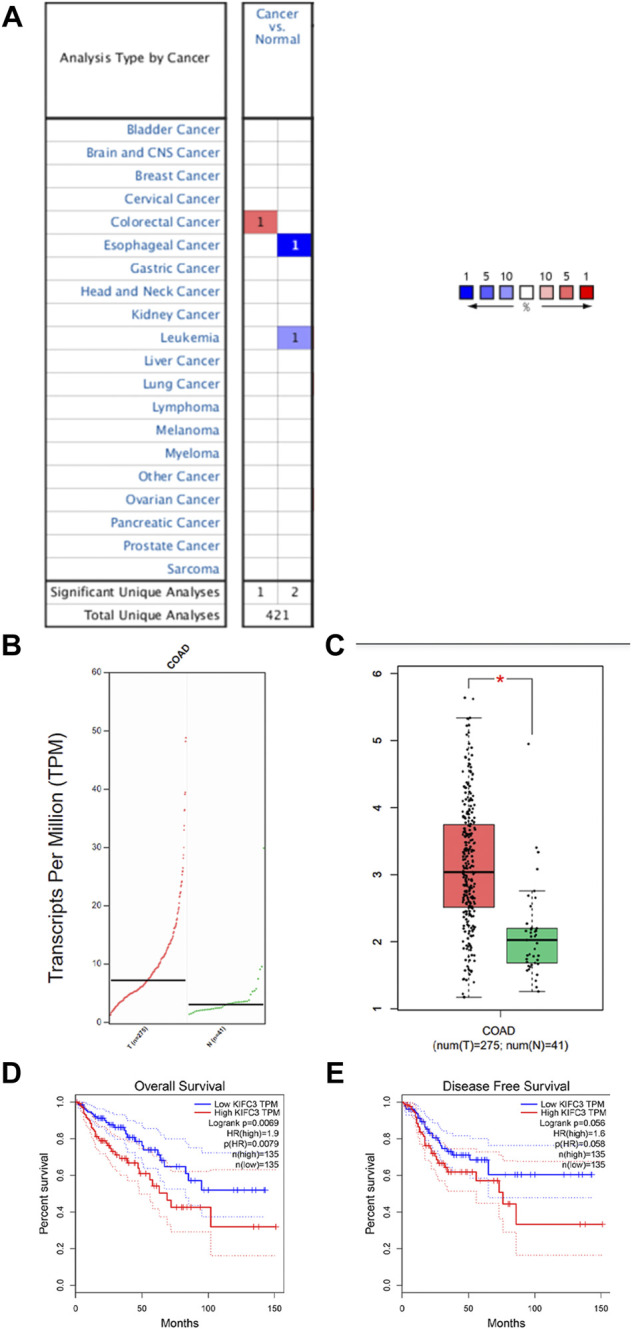
Analysis of KIFC3 mRNA expression in normal colorectal and colorectal cancer (CRC) tissues from public databases. **(A)** Data from the Oncomine databases showed that KIFC3 mRNA expression was upregulated in CRC tissues compared to normal colorectal tissues. **(B,C)** Data from the GEPIA databases showed that KIFC3 mRNA level was higher in CRC tissues compared to normal colorectal tissues. **(D,E)** Data from the GEPIA database showed that the expression level of KIFC3 could affect the OS of CRC (*p* = 0.00069), but did not affect the disease-free survival (*p* = 0.056). The OS of patients with high expression of KIFC3 was shorter than that of patients with low expression of KIFC3.

**FIGURE 2:**
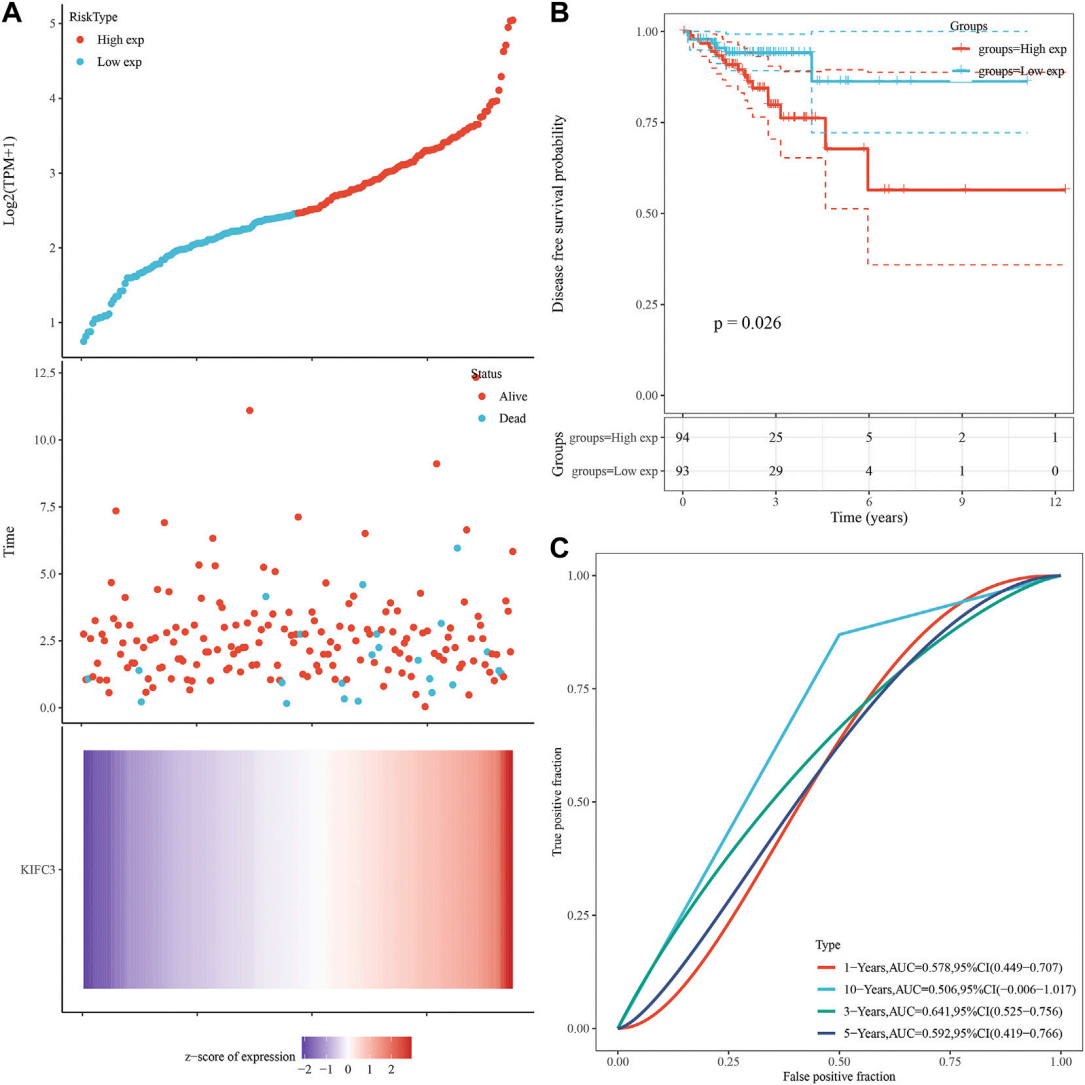
Prognostic analysis of gene signature in the ICGC database. **(A)** KIFC3 expression and survival time and survival status in ICGC data set. **(B)** Kaplan-Meier survival curve distribution of KIFC3 in ICGC data database. **(C)** ROC curve and AUC of KIFC3 at different times. The abscissas of the upper, middle and lower three graphs in Figure 2A all represent samples, and the order of the samples is consistent.

We analyzed gene expression data in the Oncomine and GEPIA databases to compare KIFC3 mRNA levels in the tumor and normal tissues from CRC patients. The CRC tissues showed significantly higher KIFC3 mRNA expression than the adjacent tissues (Figures 1A–C). Then, we use the GEPIA database to evaluate the prognostic value of KIFC3 level. Kaplan-Meier plotter survival analysis showed that the expression level of KIFC3 could affect the OS of CRC (*p* = 0.0069), but did not affect the disease-free survival (*p* = 0.056). The OS of patients in the group with high KIFC3 expression was shorter than that in the group with low KIFC3 expression, as shown in Figures 1D,E. We used ICGC database to analyze the relationship between KIFC3 gene expression and survival time and survival status. As shown in Figure 2A, the top represents the scatter plot from low to high expression level of the gene. The middle represents the scatter plot distribution of survival time and survival state corresponding to gene expression in different samples. The image at the bottom represents a heat map of the gene expression. The Kaplan-Meier survival curve distribution of KIFC3 in the ICGC database is shown in the Figure 2B, the dotted line represented the median risk score and divided the patients into low-risk and high-risk group, the disease-free survival of patients in the group with high KIFC3 expression was shorter than that in the group with low KIFC3 expression. Figure 2C display the ROC curves and AUC of the KIFC3 gene at different times. The results showed that CRC patients with higher KIFC3 mRNA levels were associated with poorer OS than the CRC patients with lower KIFC3 expression.

### Kinesin Family Member C3 and Gene Ontology Enrichment Analyses of Kinesin Family Member C3

**FIGURE 3:**
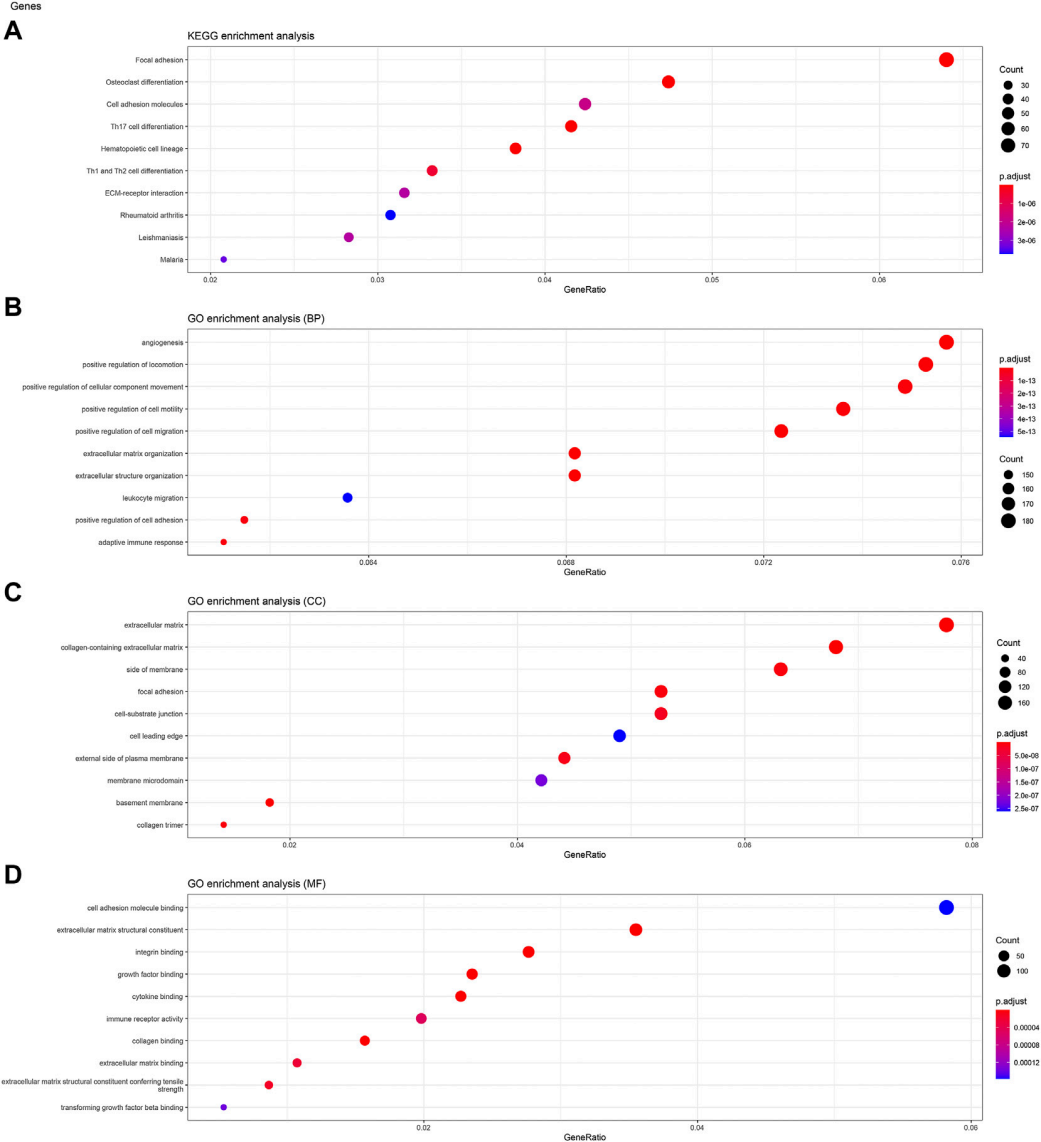
KEGG pathway and GO enrichment analyses of KIFC3-associated genes. **(A)** The bubble chart of KEGG pathway. **(B)** The bubble chart of GO enrichment in biological processes. **(C)** The bubble chart of GO enrichment in cell component. **(D)** The bubble chart of GO enrichment in molecular function.

**FIGURE 4:**
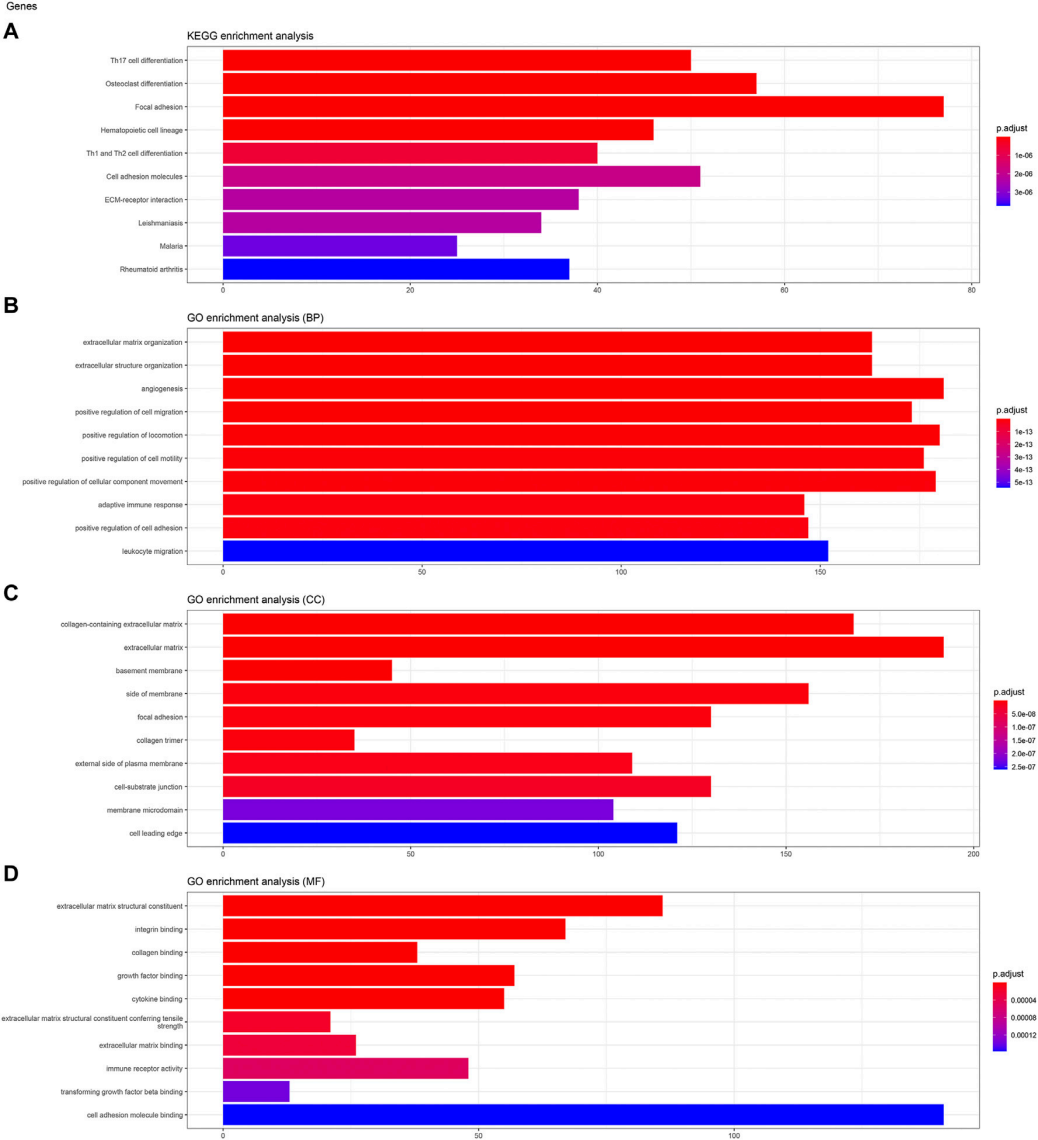
KEGG pathway and GO enrichment analyses of KIFC3-associated genes. **(A)** The bar chart of KEGG pathway. **(B)** The bar chart of GO enrichment in biological processes. **(C)** The bar chart of GO enrichment in cell component. **(D)** The bar chart of GO enrichment in molecular function.

**FIGURE 5:**
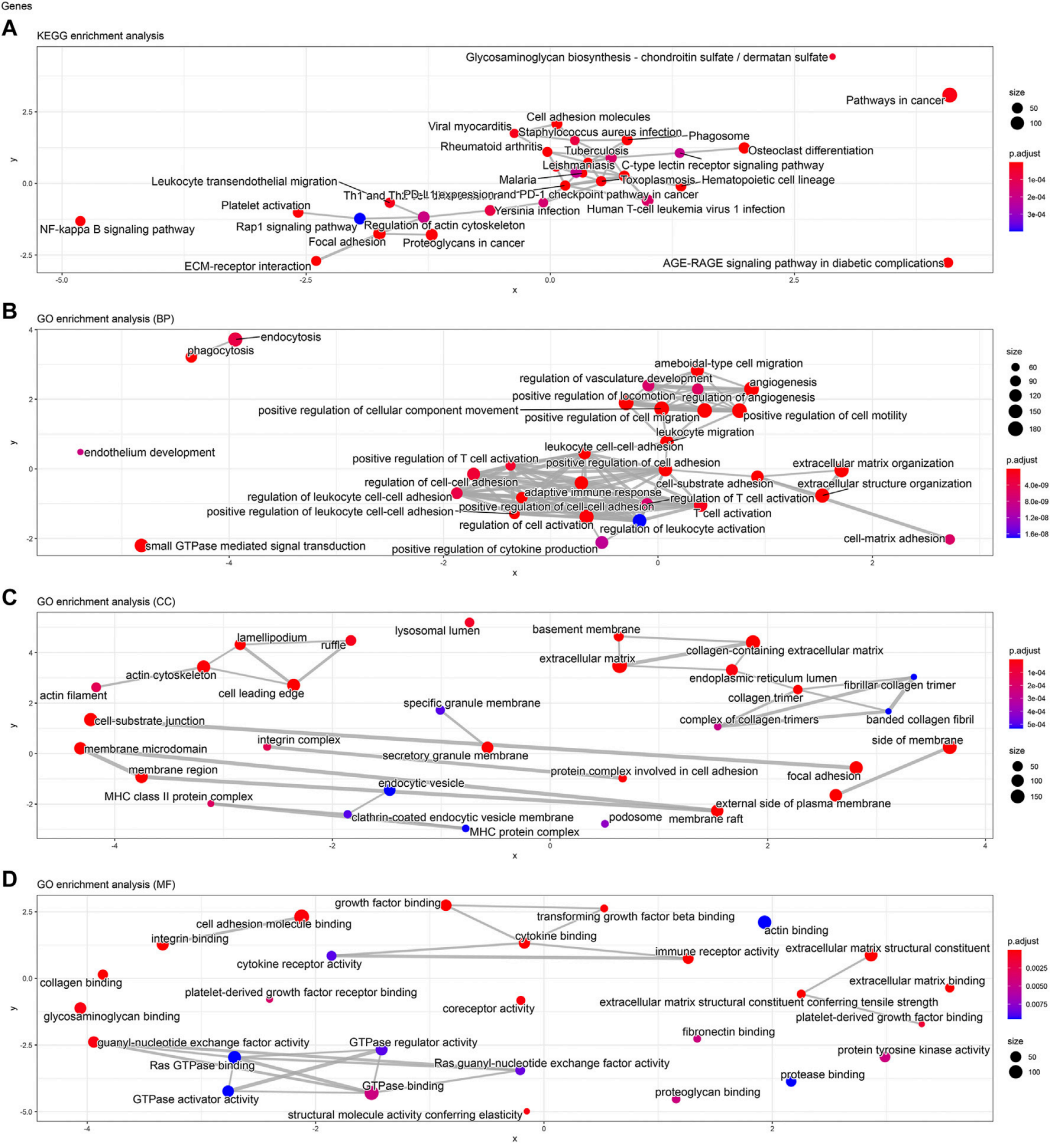
KEGG pathway and GO enrichment analyses of KIFC3-associated genes. **(A)** The network chart of KEGG pathway. **(B)** The network chart of GO enrichment in biological processes. **(C)** The network chart of GO enrichment in cell component. **(D)** The network chart of GO enrichment in molecular function.

To analyze the biological classification of KIFC3, function enrichment analyses were performed using clusterProfiler package in R. KEGG pathway analysis revealed that DEGs were mainly enriched in focal adhesion and pathways in cancer (Figures 3A, 4A, 5A). GO analysis results showed that changes in biological processes (BP) of KIFC3 were significantly enriched in angiogenesis and positive regulation of cellular component movement (Figures 3B, 4B, 5B) Changes in cell component (CC) of DEGs were mainly enriched in extracellular matrix and collagen-containing extracellular matrix (Figures 3C, 4C, 5C). Changes in molecular function (MF) were mainly enriched in extracellular matrix structural constituent and cell adhesion-molecule binding (Figures 3D, 4D, 5D). These result show, KIFC3 may be involved in the process of cell adhesion and tight connection.

### Kinesin Family Member C3 is Expressed at High Levels in Colorectal Cancer Cell Lines

**FIGURE 6:**
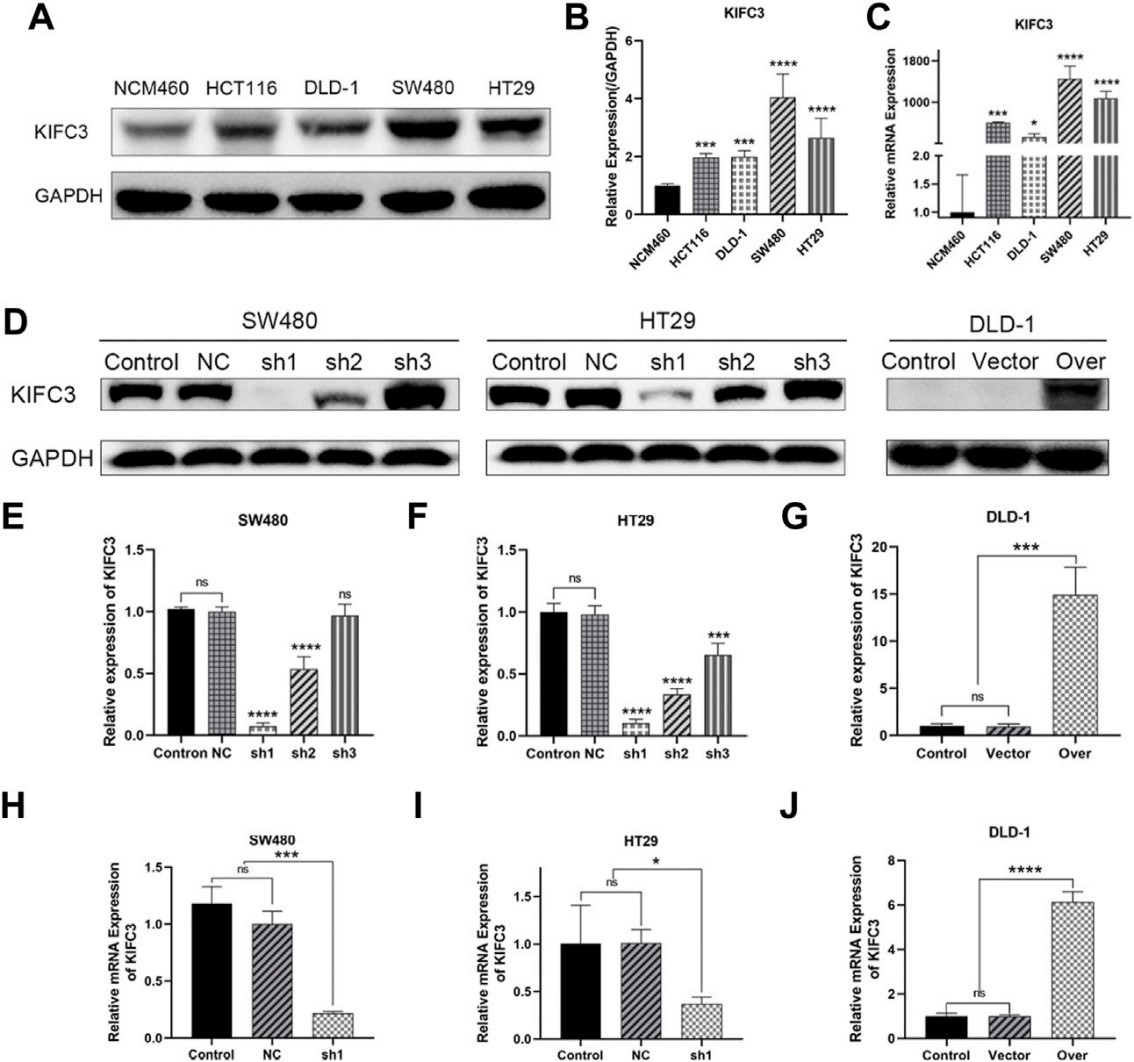
KIFC3 was expressed at higher levels in CRC cell lines. **(A–C)** Western blot and qRT-PCR analysis of KIFC3 expression in CRC cell lines (HT29, HCT116, SW480, DLD-1) and normal colon mucosal epithelial cells (NCM460), and quantification analysis results. **(D–G)** Western blot analysis of the efficiency of sh-KIFC3 and sh-NC transfection compare with Control group in SW480 and HT29 cells, and the efficiency of KIFC3-overexpress and over-Control transfection in DLD-1 cell, and quantification analysis results. **(H–J)** qRT-PCR analysis of the efficiency of the best knockdown effect in SW480 and HT29 cells, and the efficiency of KIFC3-overexpress, NC and Control transfection in DLD-1 cell. The transfected cells above are all stable transfectants. The data are presented as the mean ± standard deviation of triplicate independent experiments and were normalized to the control group. *p < 0.05; **p < 0.01; ***p < 0.001; ****p < 0.0001.

**FIGURE 7:**
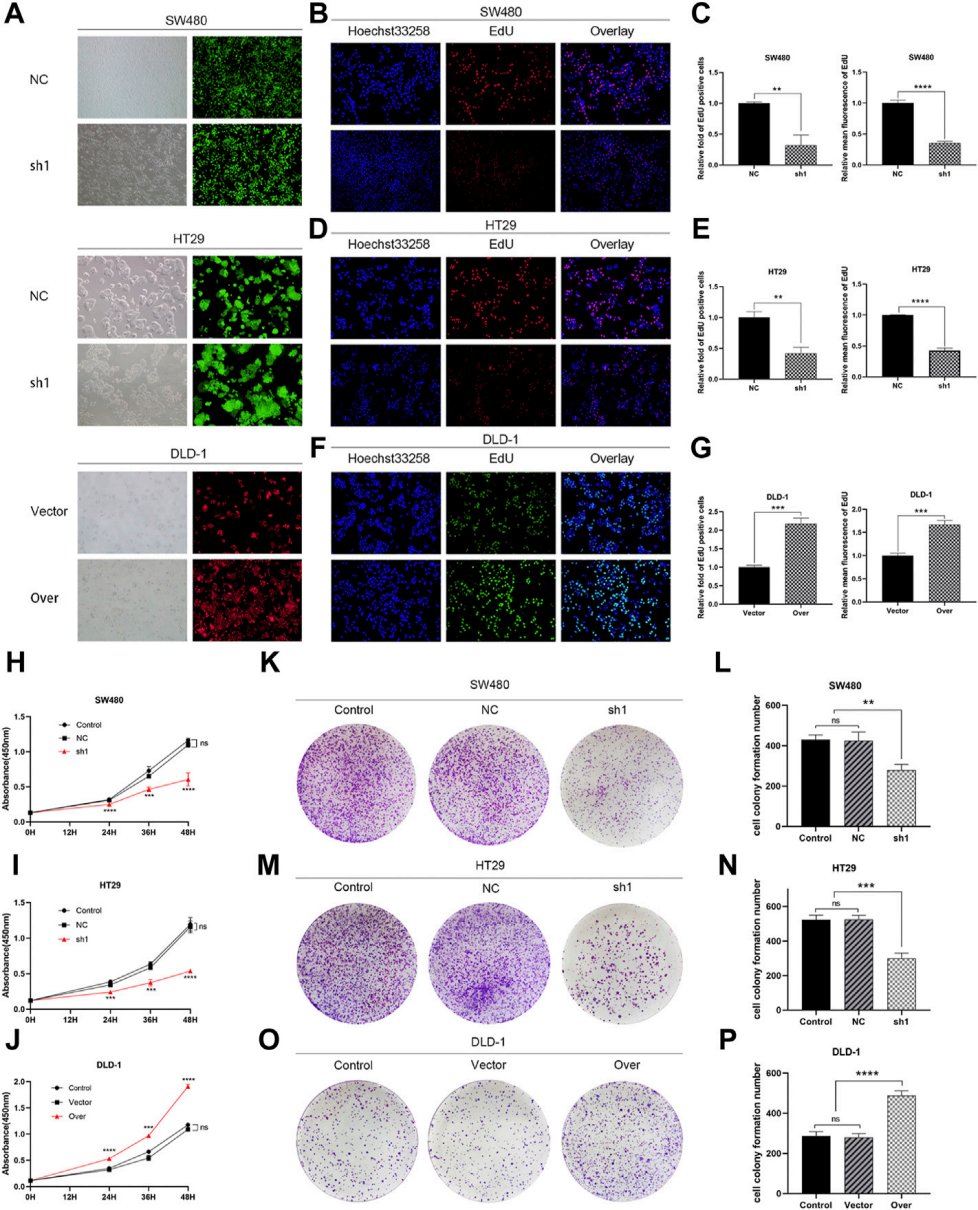
KIFC3 promotes the proliferation of CRC cells. **(A)** The transfection efficiency of SW480, HT29 and DLD-1 were observed under microscope. **(B–G)** EdU assay of KIFC3 silencing or overexpression on CRC cells’ proliferation, and quantification analysis results. **(H–J)** The effects of KIFC3 silencing or overexpression on the proliferation ability of SW480, HT29 and DLD-1 cells were measured by the CCK-8 assay. **(K–P)** Colony formation assay of KIFC3 silencing or overexpression on CRC cells’ proliferation, and quantification analysis results. The data are presented as the mean ± standard deviation of triplicate independent experiments and were normalized to the control group. *p < 0.05; **p < 0.01; ***p < 0.001; ****p < 0.0001.

The protein and mRNA levels of KIFC3 in CRC cell lines were validated by western blotting and qRT-PCR. It is confirmed that the protein levels of KIFC3 were significantly higher in CRC cells (HT29, HCT116, SW480, DLD-1) compared with that in the normal colorectal epithelium cell line NCM460 (Figures 6A,B). Likewise, the mRNA levels of KIFC3 in CRC cells were also higher than in NCM460 (Figure 6C). Among the CRC cell lines, the expression level of the KIFC3 was highest in SW480 cell followed by HT29 cell, while DLD-1 had the lowest level of KIFC3. Based on the above, we established a stable knockdown cell line of SW480 and HT29 cells, and KIFC3-overexpressing in DLD-1 cells. The picture (Figures 6D–J) shows the efficiency of knockdown or overexpression at the protein level and mRNA level. We selected the best knockdown effect for subsequent experiments, and a fluorescence microscope showed the transfection efficiency was over 95% (Figure 7A).

### Kinesin Family Member C3 Promotes Colorectal Cancer Cell Proliferation

**FIGURE 8:**
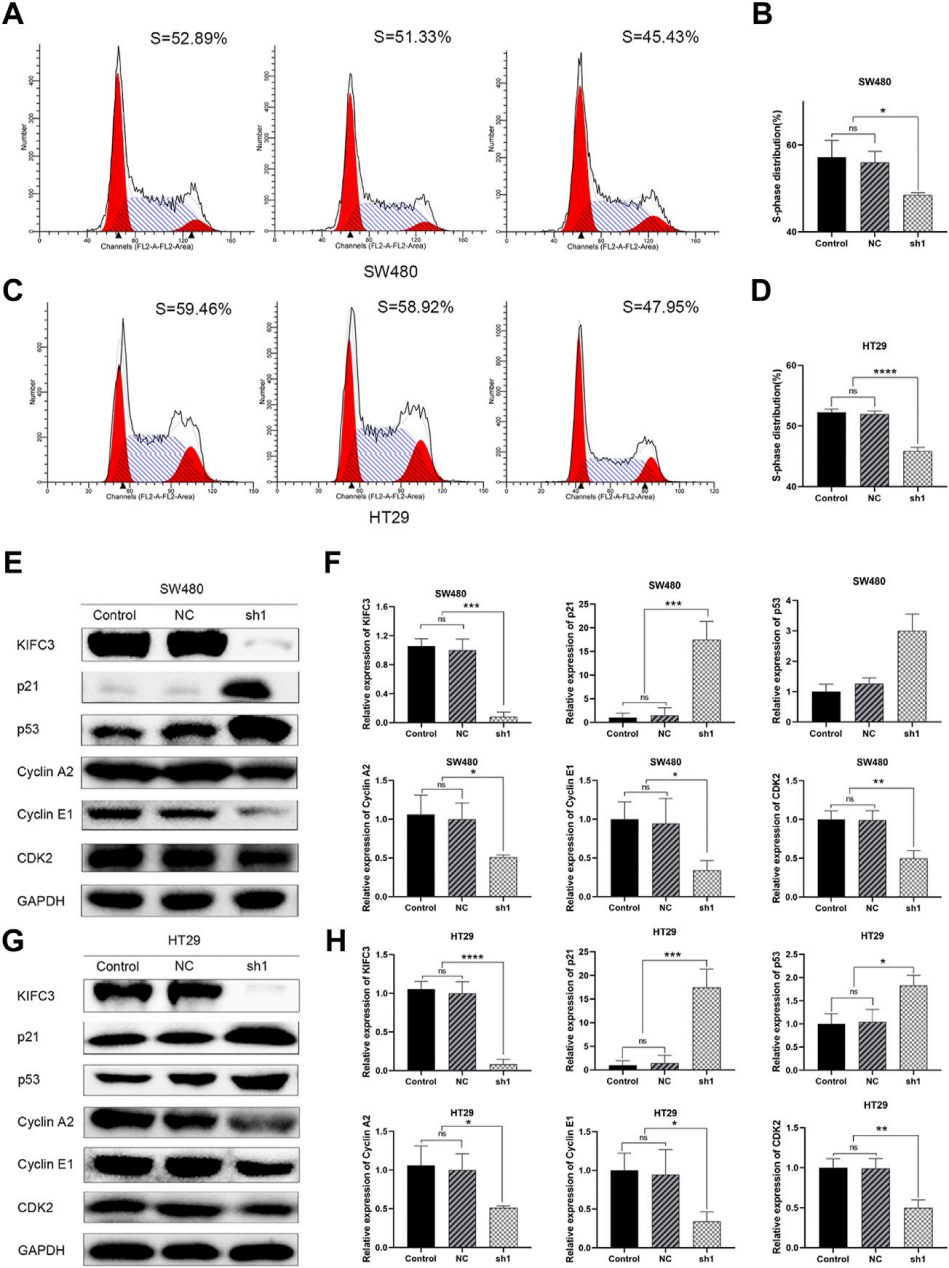
KIFC3 affects the expression of cell cycle-mediated factors. **(A–D)** Effects of KIFC3 silencing on the cell cycle distribution in SW480 and HT29 cells as showed by flow cytometry assay, and quantification analysis results. **(E–H)** Effect of KIFC3 silencing on the expression of cell cycle-mediated factors like p21, p53, Cyclin A2, Cyclin E1, and CDK2, as measured by western blot, and quantification analysis results. The data are presented as the mean ± standard deviation of triplicate independent experiments and were normalized to the control group. *p < 0.05; **p < 0.01; ***p < 0.001; ****p < 0.0001.

**FIGURE 9:**
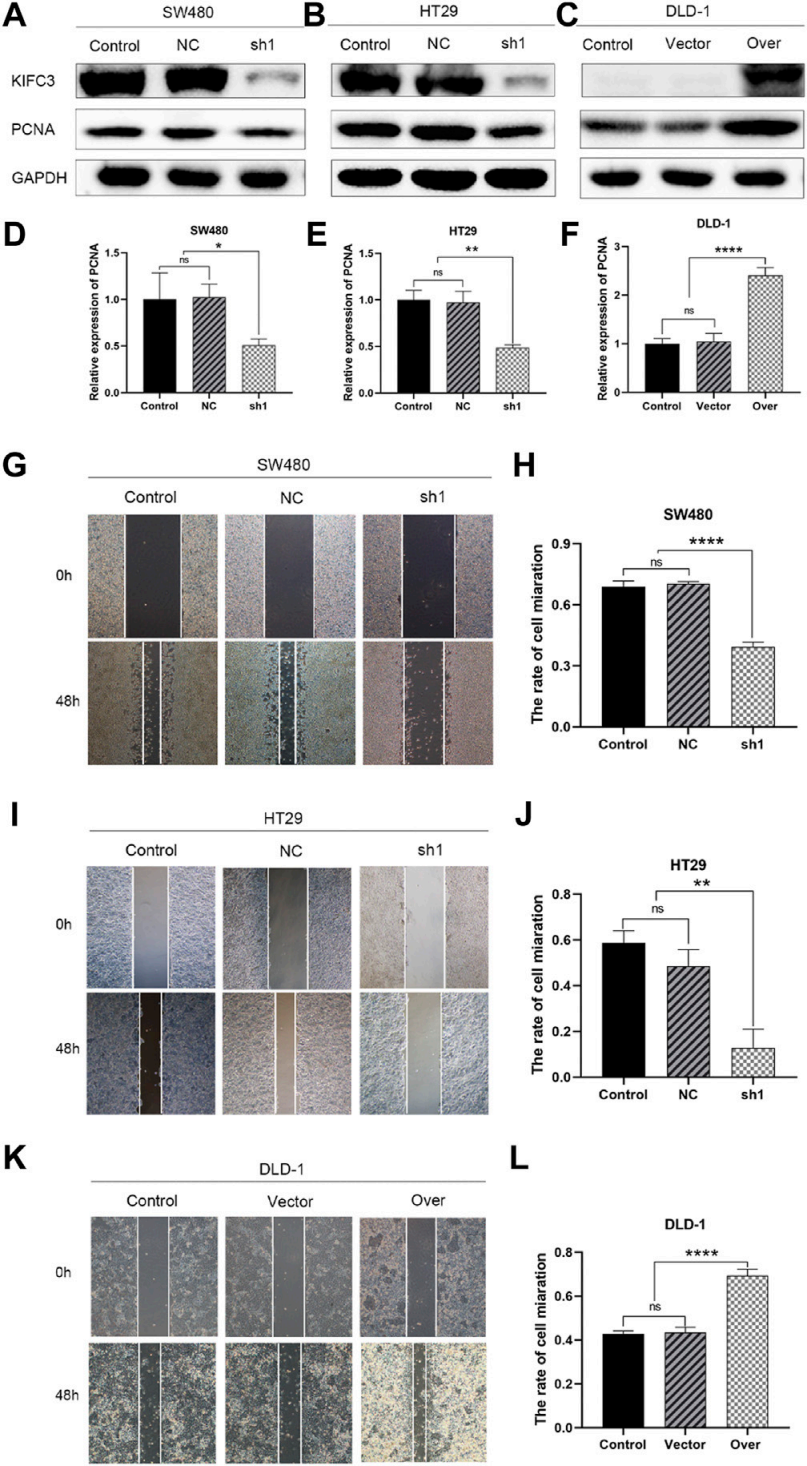
KIFC3 promotes the proliferation and migration of CRC cells. **(A–F)** The expression of PCNA in SW480, HT29 and DLD-1 cells after lentivirus vectors delivery in each group as measured by western blot, and quantification analysis results. **(G–L)** Effects of KIFC3 silencing or overexpression on the wound healing of SW480, HT29 and DLD-1 cells, and quantification analysis results. The data are presented as the mean ± standard deviation of triplicate independent experiments and were normalized to the control group. *p < 0.05; **p < 0.01; ***p < 0.001; ****p < 0.0001.

To elucidate the function of KIFC3 in cell growth, we performed EdU assay, CCK-8 assay and colony formation assay. Evaluation of EdU staining showed that depleted-KIFC3 in SW480 and HT29 cells gave rise to a decrease in the number of EdU-positive cells (Figures 7B–E), while the overexpression of KIFC3 significantly increased the number of EdU labelled cells in contrast with Control and Vector groups (Figures 7F,G). CCK-8 assay results demonstrated that the growth of KIFC3-depleted in SW480 cells was slower than the Control and NC group at 24, 36 and 48 h (Figure 7H). Similar results were shown when the assay was conducted in the depletion of KIFC3 in HT29 cells (Figure 7I). Conversely, KIFC3 overexpression group had a higher proliferation vitality than those transfected with Vector and Control groups (Figure 7J). Meanwhile, colony formation assay found that KIFC3 downregulation impaired cell viability in SW480 and HT29 cells (Figures 7K–N), whereas KIFC3 upregulation promoted cell viability in DLD-1 cells (Figures 7O,P). Due to the observed effects of KIFC3 on CRC cells growth, we assessed the cell cycle assay and found that the knockdown of KIFC3 in SW480 cell lines could increase the number of cells in G1 phase, decrease the number of cells in S phase (Figures 8A,B, Supplementary Figure S1A). Meanwhile, the knockdown of KIFC3 in HT29 cell lines could increase the number of cells in G1 phase and the number of cells in G1 phase, and decrease the number of cells in S phase (Figures 8C,D, Supplementary Figure S1B). Next, we evaluated the expression of Cyclin A2, Cyclin E1, and CDK2, which are important and tightly initiate DNA replication in S phase. We also evaluated the expression of p21 and p53. The downregulation of KIFC3 suppress the expression of Cyclin A2, Cyclin E1 and CDK2, enrich the expression of p21 and p53 (Figures 8E–H). Moreover, we added the cell cycle assay of KIFC3-overexpression cells to verify whether KIFC3 has an effect on the growth cycle of CRC. The result show that KIFC3 overexpression increase the numbers of cells in S phase, decrease the number of cells in G1 phase, further promoted the G1/S phase transition (Supplementary Figure S2). PCNA is a key marker of cell proliferation, which assists in DNA replication. We found that the cells transfected shKIFC3 had lower protein levels of PCNA than those in Control and NC, whereas the protein level of PCNA was enhanced after KIFC3 overexpression compared to the Control and Vector groups (Figures 9A–F). Those results indicate that KIFC3 was sufficient to increase the proliferation of CRC cells.

### Kinesin Family Member C3 Promotes the Migration and Invasion of Colorectal Cancer Cells

**FIGURE 10:**
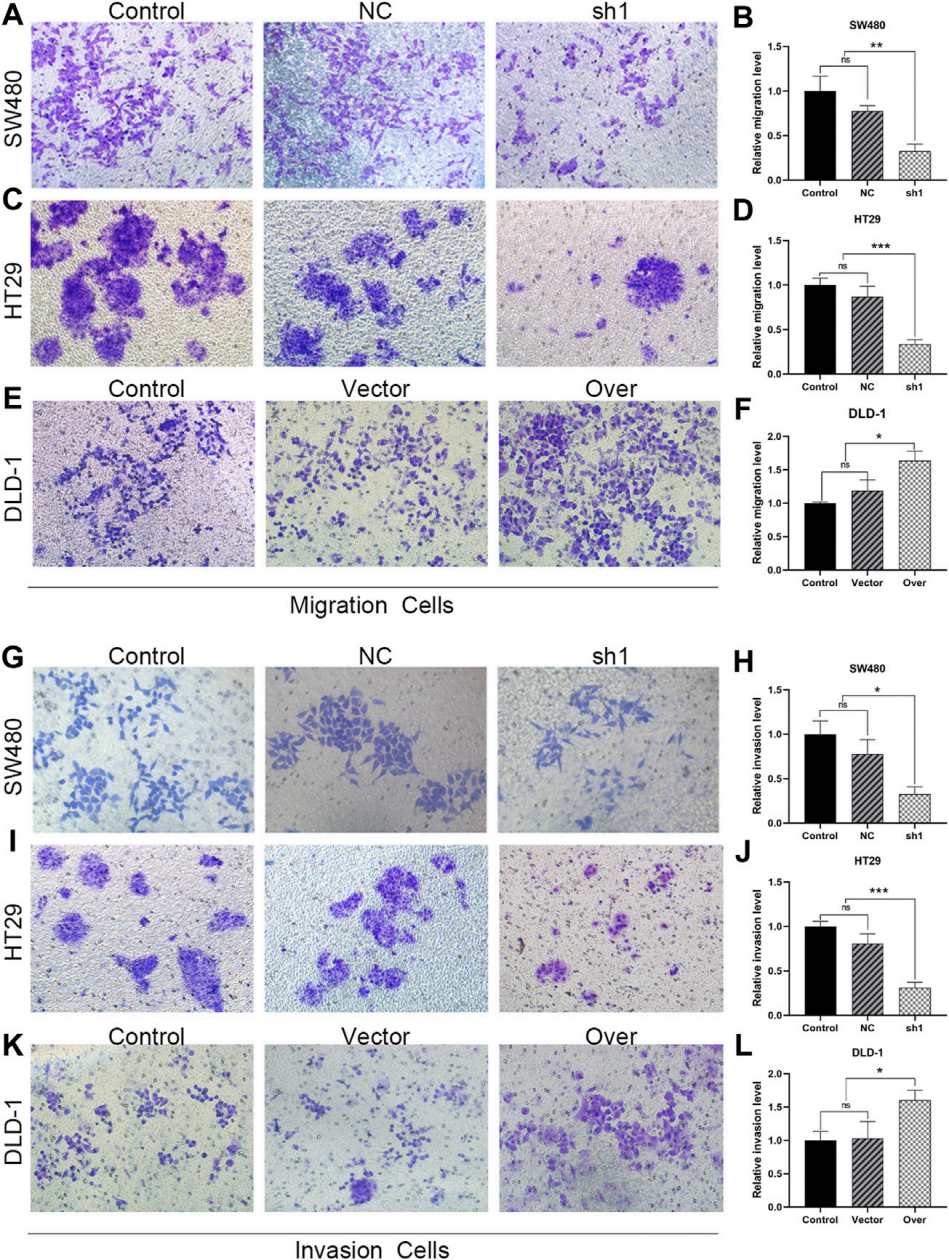
KIFC3 promotes the migration and invasion of CRC cells. **(A–F)** Effects of KIFC3 silencing or overexpression on the migration ability of SW480, HT29 and DLD-1 cells, and quantification analysis results. **(G–L)** Effects of KIFC3 silencing or overexpression on the invasion ability of SW480, HT29 and DLD-1 cells, and quantification analysis results. The data are presented as the mean ± standard deviation of triplicate independent experiments and were normalized to the control group. *p < 0.05; **p < 0.01; ***p < 0.001; ****p < 0.0001.

To test the relevance of KIFC3 on cell migration and invasion, we conducted the scratch and transwell assays. We used a wound healing assay to demonstrate the roles of KIFC3 on migration capability of CRC cells. When the cell transfected with shKIFC3, the wound-healing speed was slower than the Control and NC groups and the wound-healing speed was faster in the group of KIFC3 overexpression than the Control and Vector groups (Figures 9G–L). Moreover, the transwell assay showed similar results as wound healing assay. From the Figures 10A–D, transwell migration assays showed that the downregulation of KIFC3 significantly weakened the migratory ability in SW480 and HT29 cell lines. On the contrary, the cell migratory capability was enhanced after KIFC3 overexpression in DLD-1 cell lines (Figures 10E,F). Similarly, the invasion ability of CRC cells through the filter coated with Matrigel were reduced (Figures 10G–J). As expected, the results of transwell assay exhibited that the cell invasion rate were increased in KIFC3 overexpression groups compared with the Control and Vector groups (Figures 10K–L). The combined data demonstrated that KIFC3 may act as a regulator in promote migration and invasion in CRC cells.

### Kinesin Family Member C3 Enhances the Epithelial-to-Mesenchymal Transition Process in Colorectal Cancer Cells

**FIGURE 11:**
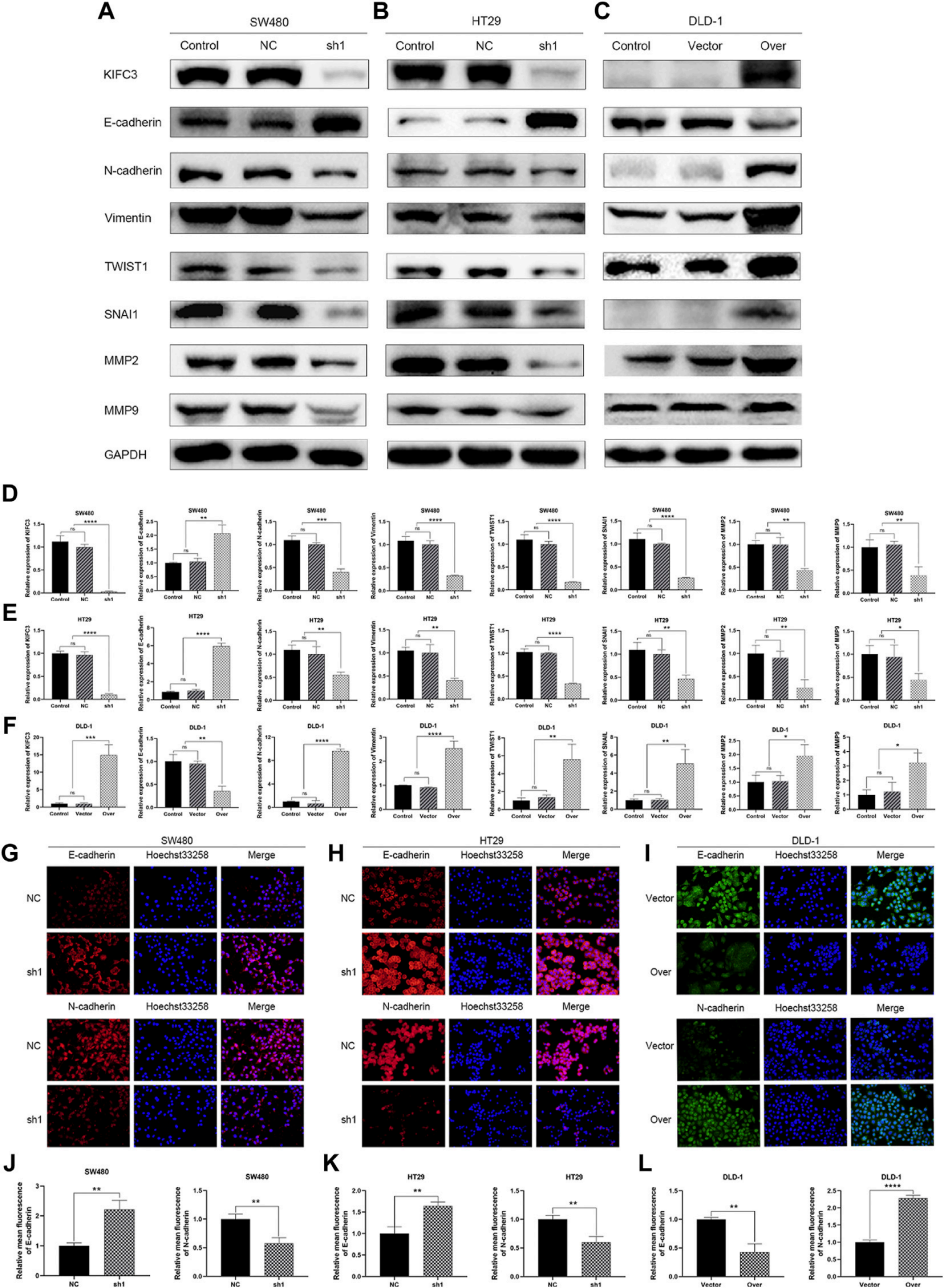
KIFC3 affected the expression of the related factors in the EMT progress. **(A–C)** Effects of KIFC3 silencing or overexpression on the expression of the related factors in the EMT progress like E-cadherin, N-cadherin, Vimentin, TWIST1, SNAI1, MMP-2, and MMP-9, as measured by western blot. **(D–F)** Quantification to the protein level of the related factors in the EMT progress. **(G–I)** Immunofluorescence staining of E-cadherin and N-cadherin. **(J–L)** Statistical analysis of the relative mean fluorescence of E-cadherin and N-cadherin. The data are presented as the mean ± standard deviation of triplicate independent experiments and were normalized to the control group. *p < 0.05; **p < 0.01; ***p < 0.001; ****p < 0.0001.

Epithelial cells may develop invasive mesenchymal stem cell-like properties through epithelial-to-mesenchymal transition (EMT). Many researches have revealed that EMT plays a critical role in tumorigenesis and metastasis. MMP-2 and MMP-9, which are proteolytic proteins of the outer membrane, are thought to be involved in cell metastasis and cell invasion. Herein, we found that KIFC3 silencing repressed the protein level of MMP-2 and MMP-9, while the KIFC3 overexpression showed the opposite results (Figures 11A–F). To further assess the role of KIFC3 in EMT, we measured the EMT-related marker through western blot analyses after KIFC3 downregulation and overexpression. The results displayed that KIFC3 silencing lead to epithelial marker E-cadherin up-regulated, while the mesenchymal markers including N-cadherin, Vimentin, SNAIL one and TWIST were down-regulated. Yet, after KIFC3 was upregulated in DLD-1 cells, the opposite results were displayed (Figures 11A–F). To further investigate the role of KIFC3 in EMT, immunofluorescence staining was conducted, and it showed dramatic upregulation of E-cadherin expression and downregulation of N-cadherin expression when KIFC3-depleted in SW480 and HT29 cells, while the opposite results were investigated when KIFC3 was upregulated in DLD-1 cells, as well (Figures 11G–L). As demonstrated by those experiments, it could be possible that the KIFC3 could enhance the EMT process in CRC cells.

### Kinesin Family Member C3 Enhances the PI3K/AKT/mTOR Signaling Pathway in Colorectal Cancer Cells

**FIGURE 12:**
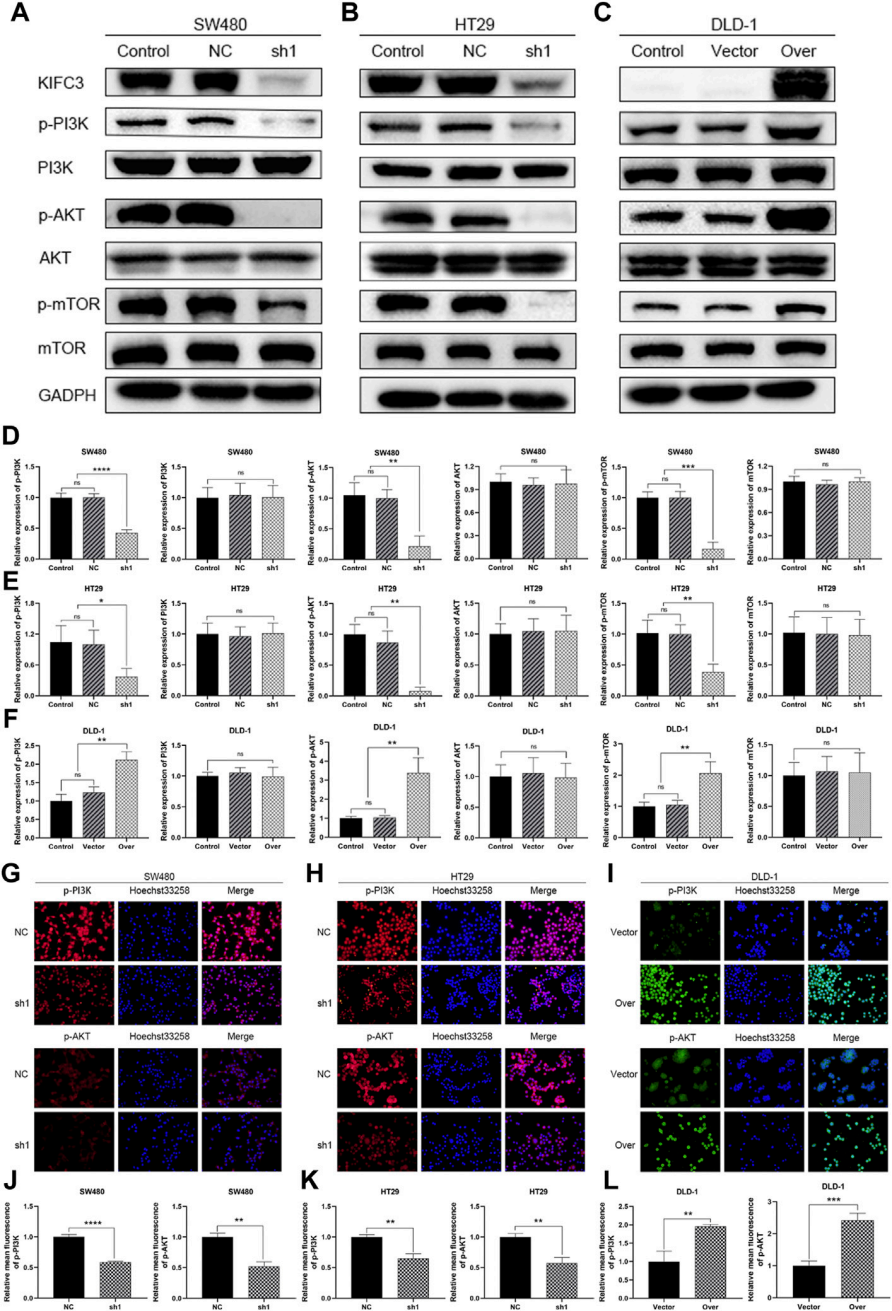
KIFC3 affected the expression of the related factors in the PI3K/AKT/mTOR pathway. **(A–C)** Effects of KIFC3 silencing or overexpression on the expression of the related factors in the PI3K/AKT/mTOR pathway like PI3K, p-PI3K, AKT, p-AKT, mTOR, and p-mTOR, as measured by western blot. **(D–F)** Quantification to the protein level of the related factors in the PI3K/AKT/mTOR pathway. **(G–I)** Immunofluorescence staining of p-PI3K and p-AKT. **(J–L)** Statistical analysis of the relative mean fluorescence of p-PI3K and p-AKT. The data are presented as the mean ± standard deviation of triplicate independent experiments and were normalized to the control group. *p < 0.05; **p < 0.01; ***p < 0.001; ****p < 0.0001.

The PI3K/AKT/mTOR signaling pathway plays a vital role in cancer which regulated cell proliferation, migration and invasion. To further elucidate the possible mechanism of KIFC3 in regulating the EMT process of CRC cells, we inspected the expression of the related proteins in PI3K/AKT/mTOR pathway. As shown by western blot assay, the protein expression levels of p-PI3K, p-AKT, and p-mTOR of KIFC3 silencing cells (SW480-shKIFC3, HT29-shKIFC3) group were notably lower compared with the Control and NC group. At the same time, the total protein levels of PI3K, AKT, and mTOR showed no changes (Figures 12A,B,D,E). As displayed in Figures 12C,F, when the KIFC3 expression was upregulated in DLD-1 cell, the protein levels of t-P13K, t-AKT and t-mTOR were unchanged, whereas the protein expression level of p-PI3K, p-AKT and p-mTOR were significantly upregulated. What is more, when KIFC3 was knocked down in SW480 and HT29 cells, immunofluorescence staining showed a downregulated expression of p-PI3K and p-AKT, the same as the western blot results, whereas the upregulation of KIFC3 reverted this phenomenon in DLD-1 cell lines (Figures 12G–L). The results above showed that the KIFC3 enhances the EMT process by activating the PI3K/AKT/mTOR signaling pathway in CRC cells.

### Loss of Kinesin Family Member C3 Inhibits Tumor Growth *in vivo*

**FIGURE 13:**
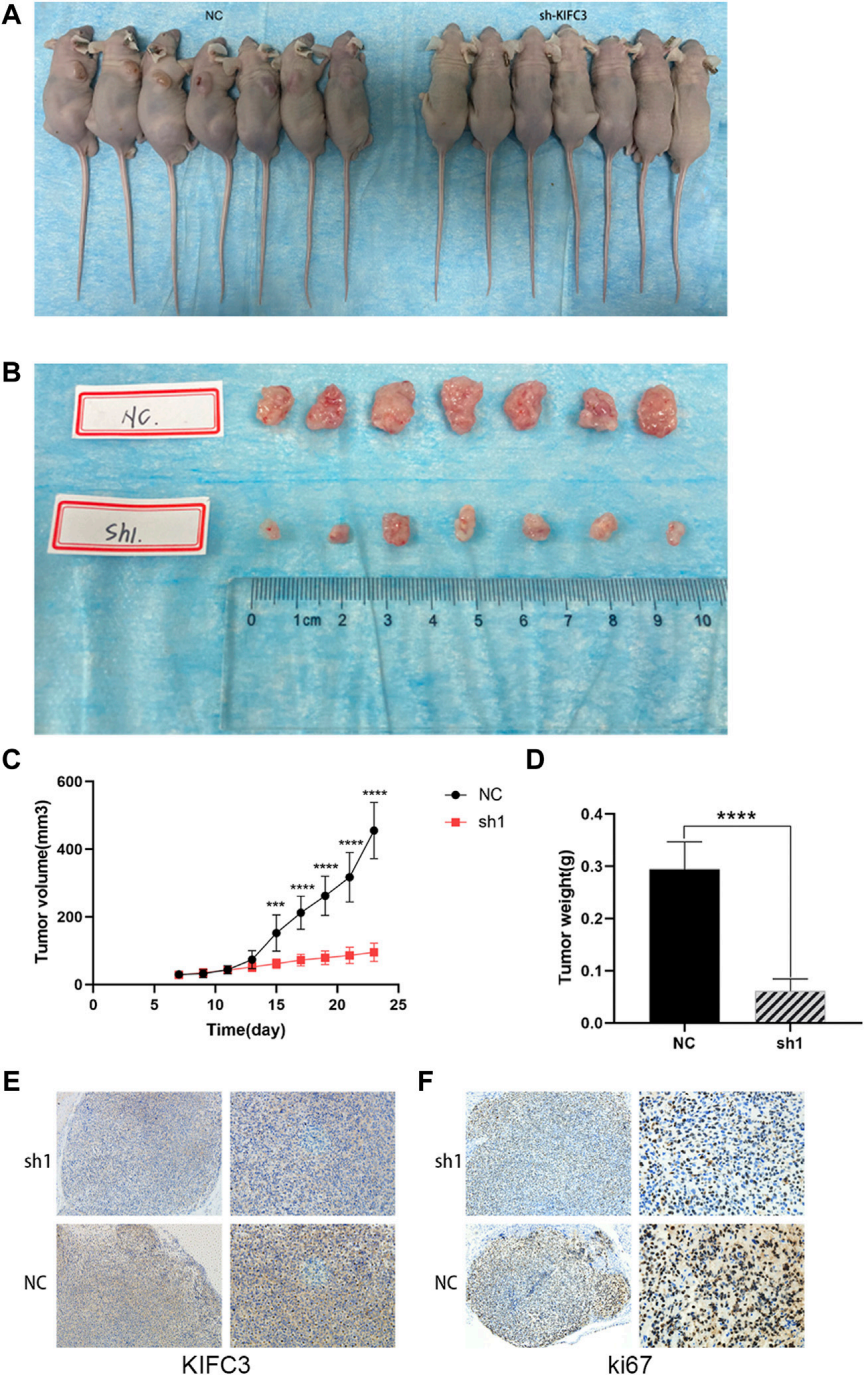
KIFC3 knockdown inhibited tumor growth and proliferation. **(A–D)** Transplanted tumor mice image, tumor xenograft image, tumor xenograft volume, tumor xenograft weight of sh-NC and sh-KIFC3 SW480 cells in nude mice. **(E,F)** Analysis of KIFC3 and Ki67 in tumor xenografts derived from sh-NC and sh-KIFC3 SW480 cells by Immunohistochemistry. The data are presented as the mean ± standard deviation of triplicate independent experiments and were normalized to the control group. *p < 0.05; **p < 0.01; ***p < 0.001; ****p < 0.0001.

Given the significant inhibition of CRC cell survival and EMT through PI3K/AKT/mTOR signaling by the knockdown of KIFC3 *in vitro*, we examined its activity *in vivo*. We established a tumor xenograft model, *via* the subcutaneous injection of the SW480 cells transfected with shKIFC3#1 or transfected with a negative control vector. One week after the tumor cell implantation, apparent subcutaneous tumor formation was observed. We then observed and measured the size of the tumor every 2 days. We found that the growth rate of tumors in the KIFC3 silencing groups were slower than that in the NC groups (Figures 13A,B). After 22 days, nude mice were sacrificed and we weighted the tumors. We found that the tumor growth rate was significantly slower in the KIFC3-depletion group than in the NC group (Figure 13C, *p* < 0.001). Then, we detected the tumor weight, and we found that the tumors in the knockdown groups were significantly smaller than those in the NC groups (Figure 13D). Furthermore, we detected the KIFC3 and ki67 expression on the tumor section. Immunofluorescence staining revealed that the xenograft tumor tissues injected with sh-KIFC3 reduced the expression of KIFC3 and ki67, indicating the decrease of CRC cell proliferation (Figures 13E,F). Collectively, these findings elucidated that the down-regulation of KIFC3 could suppress tumor growth in CRC *in vivo*.

### Inhibition of the PI3K/AKT/mTOR Pathway Attenuates the Tumorigenic Effect of Kinesin Family Member C3 on Colorectal Cancer Cells

**FIGURE 14:**
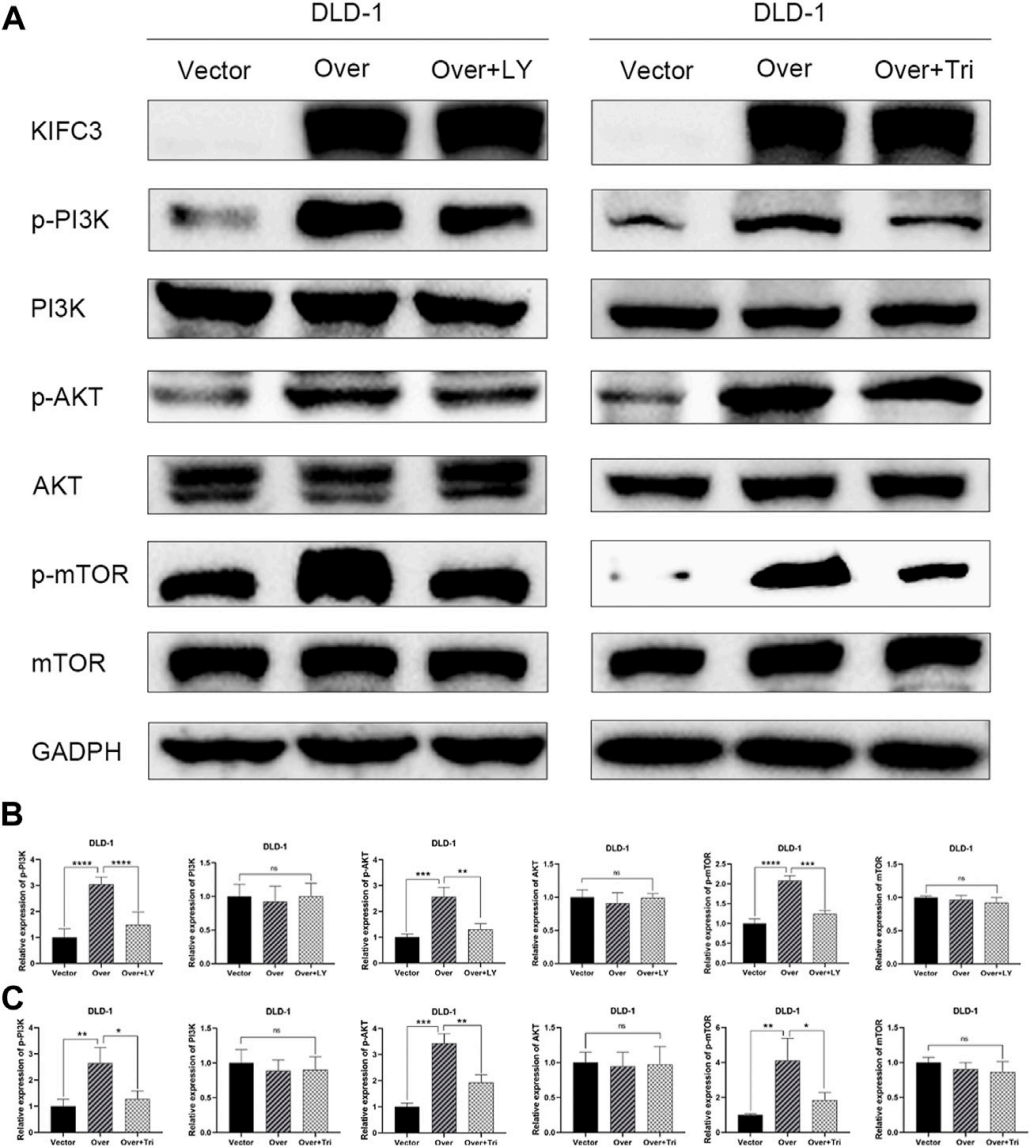
LY294002 and Triciribine attenuates the effect of KIFC3 on PI3K/AKT/mTOR pathway in CRC cells. **(A)** The expression of the related factors in the PI3K/AKT/mTOR pathway was reversed to some extent under the effect of LY294002 and Triciribine. **(B,C)** Quantification to the protein level of the related factors in the PI3K/AKT/mTOR pathway. The data are presented as the mean ± standard deviation of triplicate independent experiments and were normalized to the control group. *p < 0.05; **p < 0.01; ***p < 0.001; ****p < 0.0001.

**FIGURE 15:**
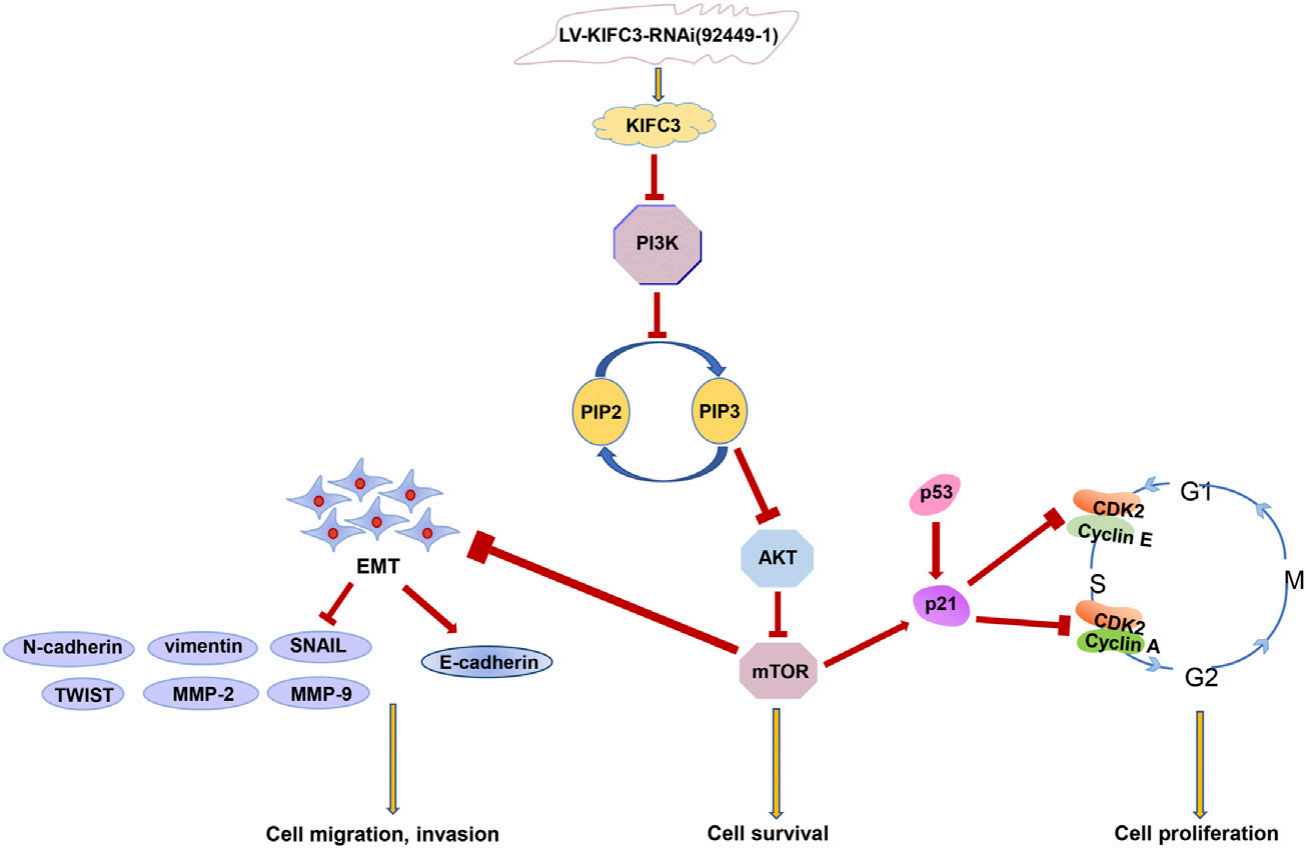
The relationship between KIFC3 and thePI3K/AKT/mTOR pathway.

To explore whether the PI3K/AKT/mTOR signaling pathway was related to KIFC3, the DLD-1 cells that transfected with KIFC3 overexpression were preincubated with LY294002 (20 μM, Selleck Chemicals, Shanghai, China) or Triciribine (5 μM, MCE, Shanghai, China). LY294002 is an inhibitor of the PI3K/AKT/mTOR pathway, whereas Triciribine is an AKT inhibitor. As represented in the Figures 14A–C, compared with the KIFC3 overexpression group, the phosphorylation levels of PI3K, AKT, and mTOR decreased after using LY294002 or Triciribine, but the total protein levels of P13K, AKT, and mTOR had no changes. Besides, LY294002 reversed the promoting effects of KIFC3 on the proliferation of CRC cells. DLD-1 cells preincubated with Triciribine shows the same trend as LY294002 (Supplementary Figure S3A). Moreover, we can find the ability of migration and invasion of CRC cells were restrained after DLD-1 cells culture with LY294002 or Triciribine (Supplementary Figures S3B,C). This finding may be attributed to the fact that KIFC3 is indeed related to the PI3K/AKT/mTOR signaling pathway (Figure 15).

## Discussion

More and more evidences indicate that KIFs play critical roles in the genesis and development of human cancers. Some KIFs are associated with malignancy and drug resistance in solid tumors (Liu et al., 2013). For example, KIF5B plays an important role in determining phenotype and aggressiveness of breast cancer (Moamer et al., 2019), high expression level of KIF11 was associated with unfavorable prognosis in clear cell renal cell carcinoma (Jin et al., 2019), KIF2C/4A/10/11/14/18B/20A/23 predict poor prognosis and promote cell proliferation in hepatocellular carcinoma (Li et al., 2020b) and KIF26A could inhibit migration and invasion (Ma et al., 2021). Aside from this, chemotherapeutic drug resistance is the main obstacle in effective tumor treatment, which hinders the efficacy of cytostatic drugs (Li et al., 2019; Tian et al., 2019; Ashar et al., 2020). In breast cancer, the overexpression of KIFC3 increased the resistance of breast cancer cells to docetaxel through opposing the microtubule stabilizing effect of docetaxel (De et al., 2009). In this study, we found that KIFC3 was expressed at higher levels in human CRC tissues and cell lines than in normal colorectal tissues and cells. That means KIFC3 may play a notable role in the occurrence and development of differential expression of the CRC.

Cellular proliferation is essential for normal development and for maintaining tissue homeostasis. Excessive proliferation of cells is closely related to the occurrence of tumors (Shi et al., 2020). Zhao et al. (2020) found that the KIF18B promotes cell proliferation in colon adenocarcinoma and tumor growth *in vitro* and *in vivo*. In our research, we found that the ability and speed of CRC cells proliferation were slower in the KIFC3-depleted groups in the EdU assay, CCK-8 and colony formation assay. The opposite results were observed in the KIFC3 overexpression groups. We further determined whether KIFC3 promotes tumor growth *in vivo*. The results showed that tumor growth rates in nude mice with KIFC3-depleted were significantly lower than in the mice of NC group. The result of the *in vivo* experiment was consistent with that of the *in vitro* experiment. Moreover, an abnormal cell cycle causes carcinogenesis and cancer progression (Kometani et al., 2020). Many studies have proved that the dysregulation of KIFs has been revealed to influence the cell cycle to cause abnormal cell growth and affect cell adhesion to promote EMT in breast, bladder, ovarian and prostate cancer. Wang et al. (2015) found that the silencing of KIF3C by shRNA inhibited epithelial-mesenchymal transition and metastasis by inhibiting TGF-β signaling and suppressed breast cancer cell proliferation through inducing G2/M phase arrest. The cell cycle assay was performed through flow cytometry. We used propidium iodide (PI), a commonly fluorescent dye that is permeable for the cell membrane and intercalates into the nucleic acid. The intensity of the signal is directly proportional to the nucleic acid content. In our study, the knockdown of KIFC3 decreased the expression of PCNA and the number of cells in S phase, whereas the overexpression of KIFC3 increased the expression of PCNA and promoted the G1/S phase transition. S phase is the phase of the cell cycle in which DNA is replicated and represents the proliferation index (Minisy et al., 2020). Cell cycle progression is co-regulated by Cyclin/Cyclin-dependent kinase (CDK) complexes. Different Cyclin-CDK complexes are involved in regulating different cell cycle transitions. Cyclin E-CDK2 plays a key role in the G1-S transition, while Cyclin A-CDK2 palys a key role in S-phase progression (Lee et al., 2013). Defects in many molecules that regulate the cell cycle also contribute to tumor progression. The factor p21, as a CDK inhibitor, is known to be mediated in the regulation of the cell cycle in cancer cells (Park W. et al., 2019). Expression of p21 is mediated by p53 and is essential for DNA-damage-induced cell cycle arrest (Sidor-Kaczmarek et al., 2017). The factor p53, as a tumor suppressor factor, suppresses cell growth, migration, and invasion. Not only does p21 serve as a downstream mediator of p53, but it also cooperates with p53 to suppress cell invasion (Kim et al., 2017). PCNA is a cofactor of DNA polymerases, which is involved in DNA synthesis during genome replication in the S phase of the cell cycle and is used as a marker reflecting the activity of cell proliferation (Lee et al., 2019). Collectively, we hypothesize that the KIFC3 increases the proliferation of CRC cells.

The misregulation of KIFs may contribute to uncontrolled cell growth, highlighting their involvement in tumorigenesis on the course of the cell cycle (Lucanus and Yip, 2018). The resulting daughter cells may exhibit cancerous behavior, including the ability of increased metastasis (Almeida and Maiato, 2018; Lucanus and Yip, 2018). The invasion and migration of tumors are the main reasons leading to the failure of tumor treatment and poor prognosis, which cause low 5-year survival rates (Zhan et al., 2020; Zhao et al., 2020). In tumorigenesis and metastasis, EMT plays a critical role. During EMT, epithelial tumor cells undergo distinct morphological and phenotypic changes, including loss of tight junctions, cell polarity and cytoskeletal reorganization, which renders cells more invasive properties and phenotypes. During metastasis, down-regulation of epithelial-associated marker proteins and up-regulation of mesenchymal marker proteins induce cell adhesion to the stroma and enhance tumor cell invasiveness. Vimentin is an intermediate filament protein and a mesenchymal marker of fibrosis. Snail as a mesenchymal marker protein affects the ability of cancer cells to invade the surrounding tissue. There is an evidence that TWIST1 upregulates vimentin expression in EMT (Meng et al., 2018). The loss of intercellular adhesion makes it easy for tumor cells to migrate and invade, eventually leading to metastatic dissemination (Liu et al., 2018). In order to penetrate into neighboring tissues and metastasize to distant organs, cancer cells require the motility and degradation of the extracellular matrix (ECM). Under this condition, certain types of ECM-degrading enzymes play a critical role in promoting the migration and subsequent metastasis of cancer cells (Pan et al., 2013). Cancer cells often release matrix metalloproteinase (MMPs), which can degrade ECM proteins. Among them, MMP-2 and MMP-9 are highly-expressed in malignant tumors, and have been proved to have participated in degradation of the ECM, a crucial component of the basal membrane, leading to cancer metastasis (Wu et al., 2019). Increased cell motility, along with the ability to digest ECM, affords cancer cells greater ability to invade tissues, leading to metastasis and diffuse tissue dissemination. Nakamura et al. (2007) established a gastric cancer cell line stably expressing KIF2C, it was found that cells transfected withKIF2C had a high rate of proliferation and increased migratory ability compared to mock-transfected cells. In our study, we found that not only the number of invading cells but also the number of migrating cells were less in the knockdown of KIFC3 group than control and NC groups. Conversely, both the number of invading and migrating cells in the KIFC3 overexpression group was higher than that in the Control and NC groups. As shown by western blot assay, KIFC3 increased the protein content of MMP-2, MMP-9 and mesenchymal associated markers, decreased the protein level of associated markers. These findings may be attributed to the fact that KIFC3 serves a key role in facilitating tumor cell invasiveness and metastasis by regulating MMP-2, MMP-9 and EMT-associated marker proteins expression.

The PI3K/AKT/mTOR pathway not only plays a vital role in physiologically cell biology but has also been identified as a growing target for tumor therapy (Dey et al., 2019; Reddy et al., 2019; Wang et al., 2019; Mannella et al., 2021). Increasing researches have reported that the PI3K/AKT/mTOR molecular signaling pathways play an essential role in the development and progression of CRC which regulate cell survival, growth, proliferation, angiogenesis, invasion, migration and glucose metabolism (Park S. H. et al., 2019; Deng et al., 2019; Jin et al., 2020; Smit et al., 2020). PI3K belongs to lipid kinases family that activated by a large number of RTKs, once stimulated, can produce phosphotidylinositol-4,5-bisphosphate (PIP2) and phosphatidylinositol-3,4,5- trisphosphate (PIP3). PIP3 plays a key role in cell growth and survival by activating downstream signaling pathways through AKT. AKT, also called as protein kinase B (PKB), when the double-phosphorylated AKT separates from the membrane, thus resulting in the activation of mTOR complex, which activates the translation of proteins, enhances cell growth, promotes cell proliferation and cell metabolism. Additionally, the complex of mTOR could exert its positive feedback and then enhance the basal phosphorylation of AKT. It has been reported that Akt plays a critical role on post-transcription of Vimentin, which is involved in the EMT process (Zhuang et al., 2012; Treesuwan et al., 2018). Also, researchers found that KIFs help to cross-link vimentin in microtentacles, which are important for cancer metastasis (Lucanus and Yip, 2018). Current researchers showed that activation of the PI3K/AKT/mTOR pathway could facilitate the process of EMT, thus increasing the metastatic ability of tumor cells. In this study, we evaluated the effects of KIFC3 on the PI3K/AKT/mTOR pathway in CRC cells. Our research data indicate that the phosphorylation levels of PI3K, AKT and mTOR were significantly increased in KIFC3-overexpressing cells, while t-PI3K, t-AKT, and t-mTOR levels had no change. The opposite trend was shown in the KIFC3-depletion groups. In order to further elucidate whether the effect of KIFC3 on CRC cells is related to PI3K/AKT/mTOR pathway, LY294002 (an inhibitor of PI3K/AKT/mTOR pathway) and Triciribine (an inhibitor of AKT) was used to treat KIFC3-overexpressing cells individually. The results showed that LY294002 and Triciribine significantly attenuated the tumor-promoting effect of KIFC3 on CRC cells. We speculate that overexpress of KIFC3 may promote the proliferation, migration and invasion on CRC cells, by activating the PI3K/AKT/mTOR pathway.

In summary, our study presented here demonstrates, for the first time, that an increased KIFC3 expression is correlated with the proliferation, migration and invasion of CRC cells through regulating the EMT process *via* the PI3K/AKT/mTOR signal transduction pathway. Therefore, we propose that KIFC3 is an important protein in the development of CRC. KIFC3 may be a promising biomarker that provides a new perspective into human CRC treatment, as well as targeting KIFs therapy seems to be a promising anti-cancer strategy.

## Data Availability

The original contributions presented in the study are included in the article/Supplementary Material, further inquiries can be directed to the corresponding author.

## Abbreviations

CRC, colorectal cancer; KIFs, Kinesin Superfamily Proteins; KIFC3, kinesin family member C3; PI3K, phosphatidylinositol 3-kinase; p-PI3K, phosphorylated phosphatidylinositol 3-kinase; t-PI3K, total phosphatidylinositol 3-kinase; p-AKT, phosphorylated Akt; mTOR, mammalian target of rapamycin; p-mTOR, phosphorylated mammalian target of rapamycin; PCNA, proliferating cell nuclear antigen; MMP, matrix metalloproteinase; GAPDH, glyceraldehyde-3-phosphate dehydrogenase.
