# Supplementary figures and images for: KIFC3 Promotes Proliferation, Migration, and Invasion in Colorectal Cancer via PI3K/AKT/mTOR Signaling Pathway
Source: Front Genet. 2022 Jun 22;13:848926. doi: 10.3389/fgene.2022.848926 (PMC9257096; doi:10.3389/fgene.2022.848926)

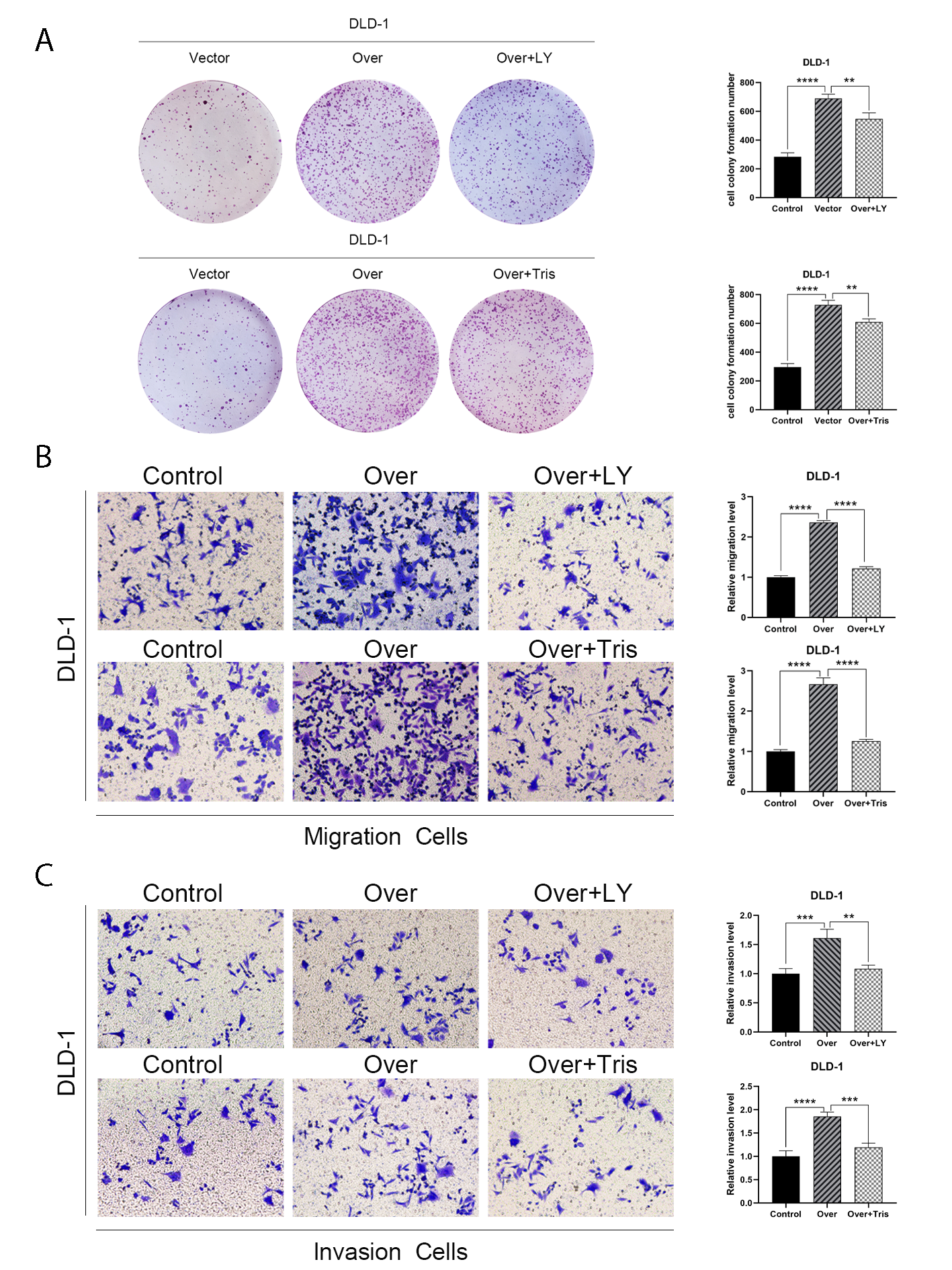

Supplement: Supplementary file 1 [file Image3.TIF]

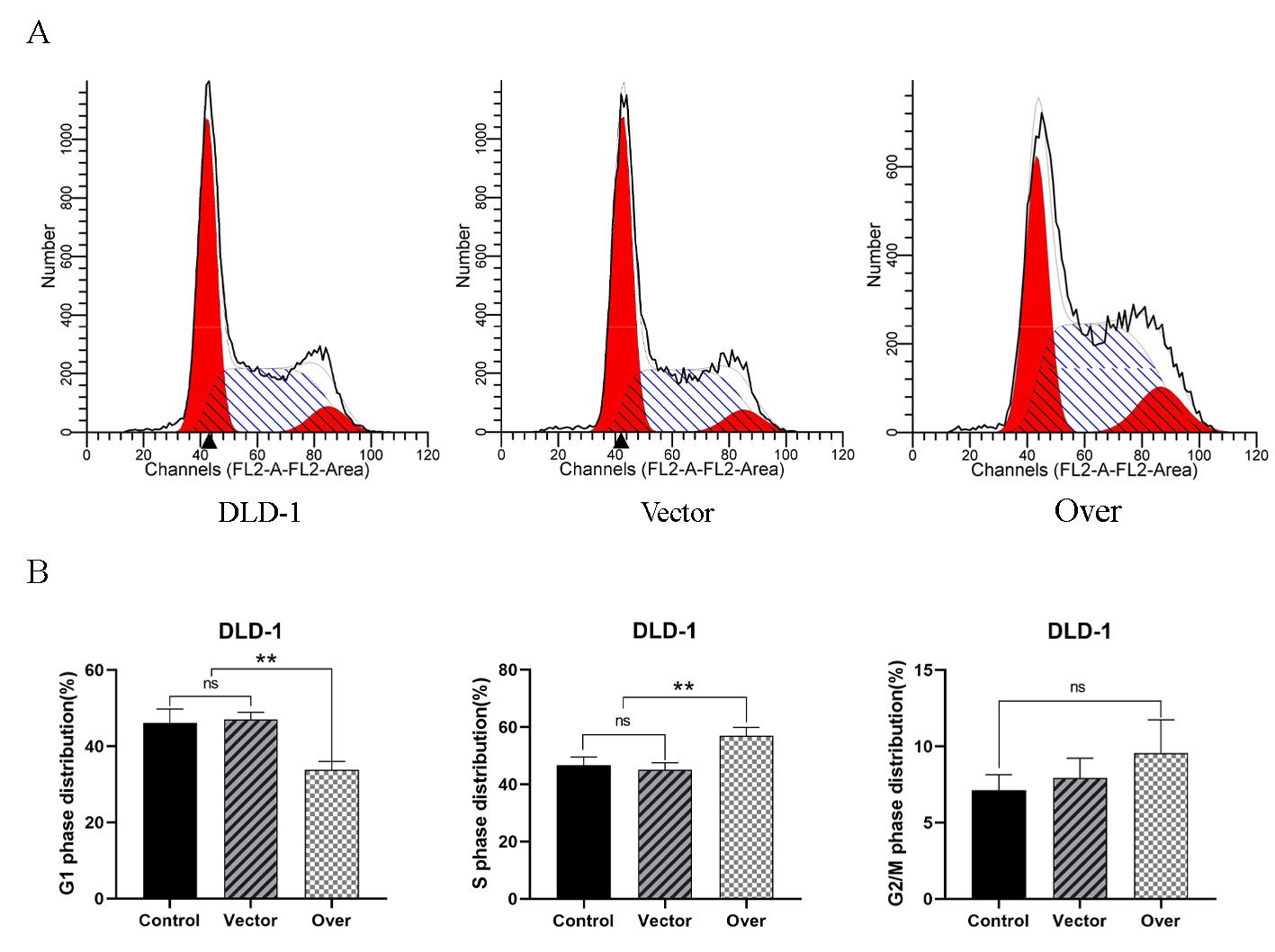

Supplement: Supplementary file 2 [file Image2.TIF]

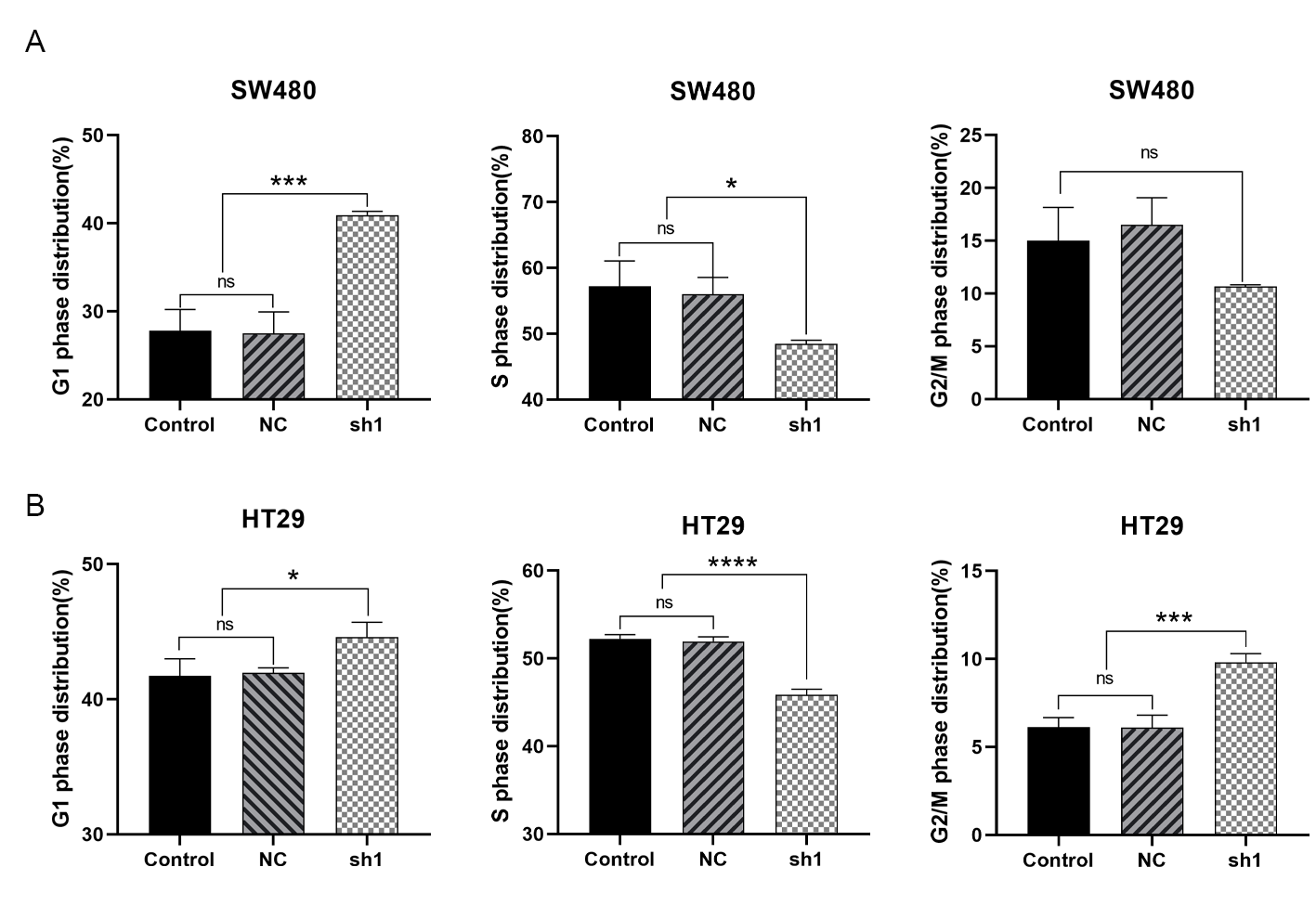

Supplement: Supplementary file 3 [file Image1.TIF]
