# Supplementary material for: KIFC3 Promotes Proliferation, Migration, and Invasion in Colorectal Cancer via PI3K/AKT/mTOR Signaling Pathway
Source: Front Genet. 2022 Jun 22;13:848926. doi: 10.3389/fgene.2022.848926 (PMC9257096; doi:10.3389/fgene.2022.848926)
Supplement: Supplementary file 4 [file DataSheet1.docx]

Supplementary Material

# Supplemental Results and Figures

Compared with the Control and NC group, the number of cells in G1 phase increased when KIFC3 was depleted in SW480 cells (27.79±2.43% vs. 27.49±2.45% vs. 40.93±0.42, Control vs. NC vs. sh1, *p* < 0.001, **Supplementary Figure 1A**). Compared with the Control and NC group, the number of cells in S phase decreased when KIFC3 was depleted (57.19±3.86% vs. 56.00±2.56% vs. 48.49±0.52, Control vs. NC vs. sh1, *p* < 0.05, **Supplementary Figure 1A**). Besides, the number of cells in G2/M phase has no significance when KIFC3 was depleted (15.01±3.15% vs. 16.51±2.57% vs. 10.67±0.15, Control vs. NC vs. sh1, *p*>0.05, **Supplementary Figure 1A**). Compared with the Control and NC group, the number of cells in G1 phase increased when KIFC3 was depleted in HT29 cell (41.75±1.26% vs. 41.97±0.37% vs. 44.59±1.11, Control vs. NC vs. sh1, *p* < 0.05, **Supplementary Figure 1B**). Besides, compared with the Control and NC group, the number of cells in S phase and G2/M phase decreased when KIFC3 was depleted in HT29 cell (S phase: 52.24±0.47% vs. 51.94±0.53% vs. 45.86±0.62, Control vs. NC vs. sh1, *p* < 0.0001; G2/M phase: 6.11±0.56% vs. 6.10±0.71% vs. 9.82±0.49, Control vs. NC vs. sh1, *p*<0.001, **Supplementary Figure 1B**).


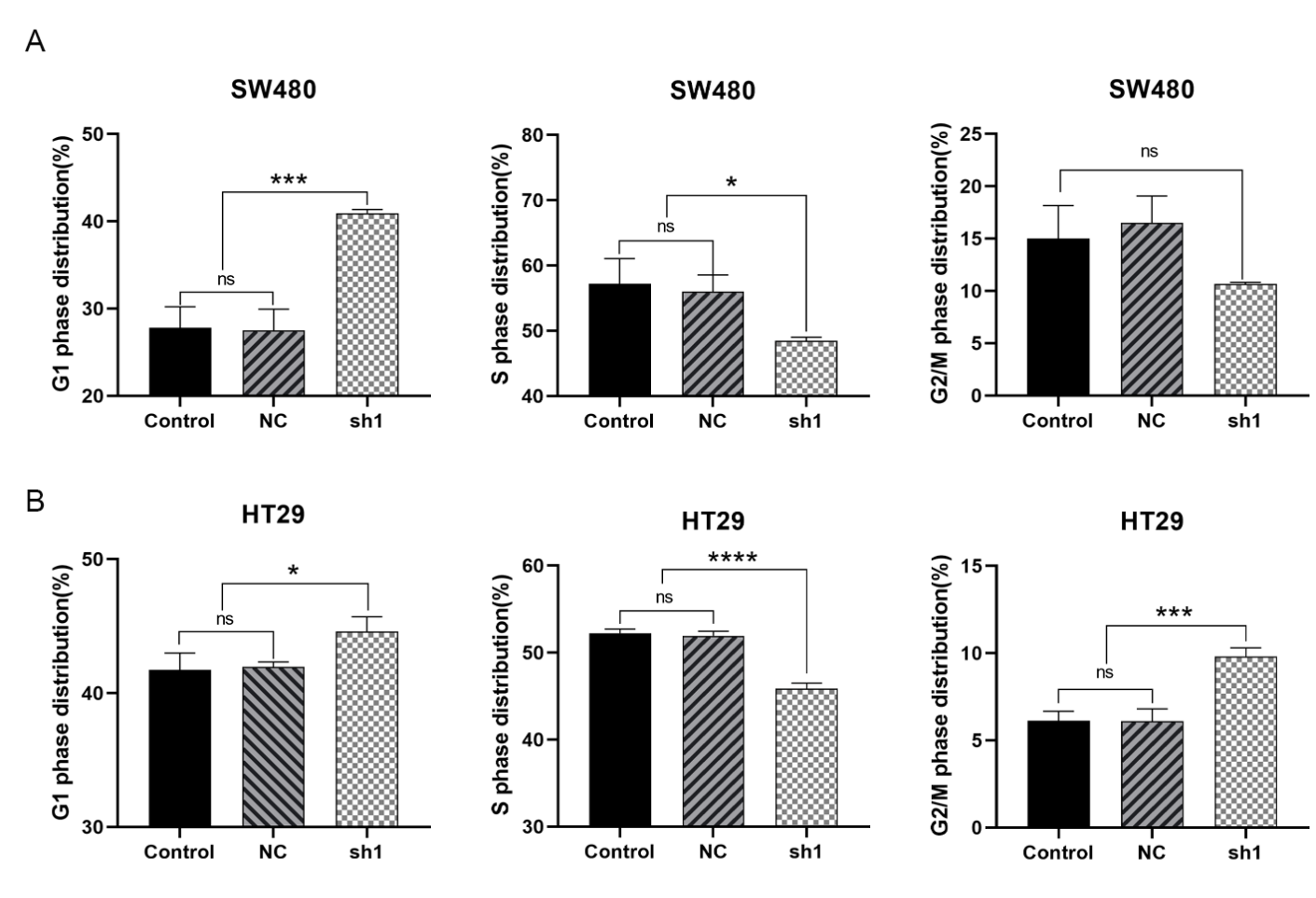


**Supplementary Figure 1.** The effect of KIFC3 down-regulation on cell cycle. (A) Quantitative analysis of the effect of deletion of KIFC3 in SW480 cells on cell cycle. (B) Quantitative analysis of the effect of deletion of KIFC3 in HT29 cells on cell cycle. The data are presented as the mean ± standard deviation of triplicate independent experiments and were normalized to the control group. * *p*< 0.05; ** *p* < 0.01; *** *p* <0.001; *****p* < 0.0001.

Compared with the Control and Vector group, the number of cells in G1 phase significantly decreased in the overexpression group (46.11±3.65% vs. 47.03±1.91% vs. 33.79±2.232%, Control vs. Vector vs. Over, *p* < 0.01, **Supplementary Figure 2B**). Compared with the Control and Vector group, the number of cells in S phase significantly increased in the overexpression group (46.76±2.77% vs. 45.06±2.52% vs. 56.96±2.94%, Control vs. Vector vs. Over, *p* < 0.01, **Supplementary Figure 2B**). Compared with the Control and Vector group, the number of cells in G2/M phase has no significance (7.13±1.00% vs. 7.92±1.31% vs. 9.57±2.17%, *p*>0.05, **Supplementary Figure 2B**).


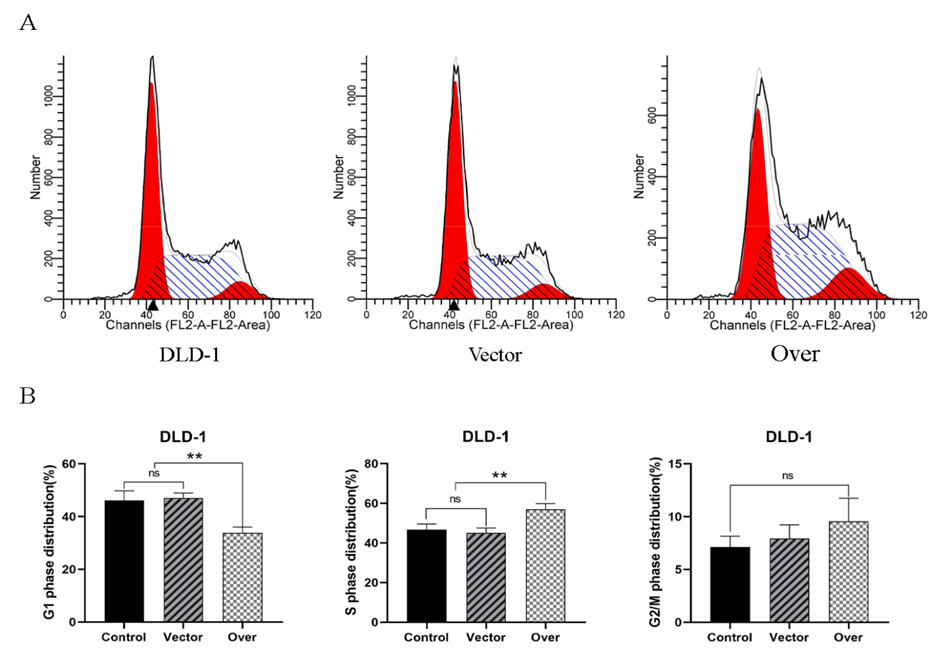


**Supplementary Figure 2.** The results of flow cytometry assay in DLD-1 cells. (A) Effects of KIFC3 overexpression on the cell cycle. (B) Quantitative analysis results of KIFC3 overexpression on the cell cycle. The data are presented as the mean ± standard deviation of triplicate independent experiments and were normalized to the control group. * *p*< 0.05; ** *p* < 0.01; *** *p* <0.001; *****p* < 0.0001.

Compared with the Vector group, a higher proliferation vitality could be seen in the overexpression group. However, when preincubated with LY294002 or Triciribine, the cell viability impaired (LY294002: 284.3±26.8 vs. 689.0±30.1 vs. 546.0±43.5, *p* < 0.01. Triciribine: 296.3±23.8 vs. 727.3±32.8 vs. 609.3±21.95, *p* < 0.01, **Supplementary Figure 3A**). Compared with the Vector group, the migration vitality was higher in the overexpression group. When preincubated with LY294002 or Triciribine, the migration viability impaired (LY294002: 1.000±0.039 vs. 2.362±0.047 vs. 1.215±0.047, *p* < 0.0001. Triciribine: 1.000±0.045 vs. 2.667±0.160 vs. 1.255±0.043, *p* < 0.0001, **Supplementary Figure 3B**). Besides, the invasion vitality was higher in the overexpression group. When preincubated with LY294002 or Triciribine, the invasion viability impaired (LY294002, 1.000±0.087 vs. 1.611±0.151 vs. 1.084±0.063, *p* < 0.01, Triciribine, 1.000±0.119 vs. 1.858±0.090 vs. 1.193±0.089, *p* < 0.001, **Supplementary Figure 3C**).


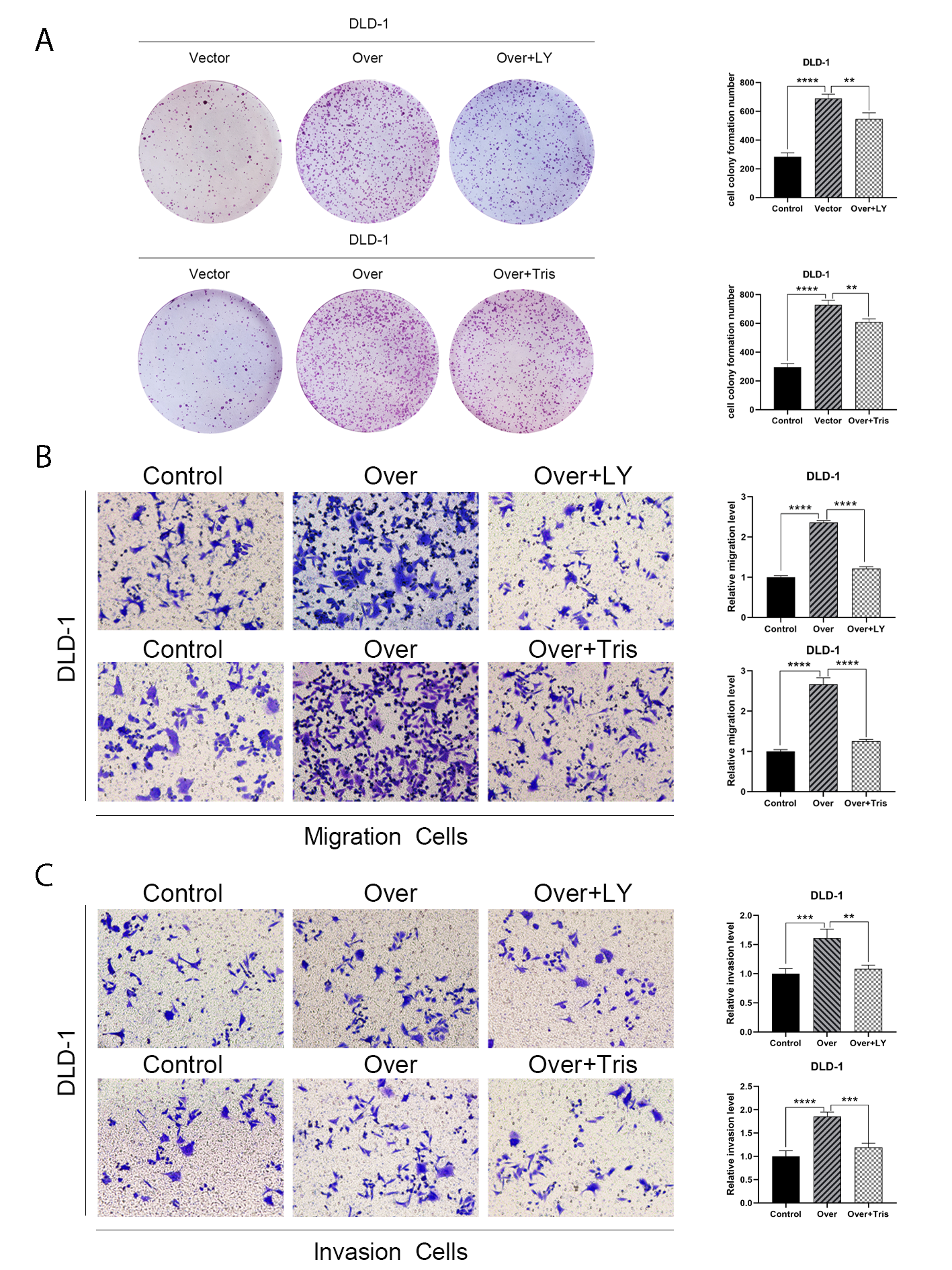


**Supplementary Figure 3.** LY294002 and Triciribine attenuates the effect of KIFC3 on proliferation, migration and invasion in CRC cells. (A) LY294002 and Triciribine attenuates the effect of KIFC3 on proliferation ability of CRC cells, and quantitative analysis results. (B-C) LY294002 and Triciribine attenuates the effect of KIFC3 on the migration and invasion ability of CRC cells, and quantitative analysis results. The data are presented as the mean ± standard deviation of triplicate independent experiments and were normalized to the control group. * *p*< 0.05; ** *p* < 0.01; *** *p* <0.001; *****p* < 0.0001.
